# The Current Position of Postoperative Radiotherapy for Salivary Gland Cancer: A Systematic Review and Meta-Analysis

**DOI:** 10.3390/cancers16132375

**Published:** 2024-06-28

**Authors:** Jingbo Wang, Ji Eun Moon, Xin Guo, Jiaqi Yu, Junlin Yi, Sun Hyun Bae

**Affiliations:** 1 Department of Radiation Oncology, National Cancer Center/National Clinical Research Center for Cancer/Cancer Hospital, Chinese Academy of Medical Sciences and Peking Union Medical College, No. 17 Panjiayuannanli, Chaoyang District, Beijing 100021, China; wangjingbo201001@163.com (J.W.); amyg1995@126.com (X.G.); jqyu95@163.com (J.Y.); 2 Department of Biostatistics, Soonchunhyang University College of Medicine, Bucheon, 170 Jomaru-ro, Wongmi-gu, Bucheon-si 14584, Gyeonggi-do, Republic of Korea; moon6188@schmc.ac.kr; 3 Department of Radiation Oncology, Soonchunhyang University College of Medicine, Bucheon, 170 Jomaru-ro, Wongmi-gu, Bucheon-si 14584, Gyeonggi-do, Republic of Korea

**Keywords:** postoperative radiotherapy, radiotherapy, salivary gland cancer

## Abstract

**Simple Summary:**

Given the low incidence, heterogeneous behavior, and diverse anatomical sites of salivary gland cancer (SGC), there are a limited number of clinical studies on its management. This is the first systematic review and meta-analysis to report the cumulative evidence on postoperative radiotherapy (PORT) for SGC of the head and neck. Based on 2962 patients with SGC from 26 studies, this study demonstrated the long-term survival and toxicities of PORT as a local treatment modality for SGC. Considering the suboptimal disease-free survival and distant metastasis-dominant recurrent patterns, however, an intensified treatment strategy is needed.

**Abstract:**

Background: Because of the rarity, heterogeneous histology, and diverse anatomical sites of salivary gland cancer (SGC), there are a limited number of clinical studies on its management. This study reports the cumulative evidence of postoperative radiotherapy (PORT) for SGC of the head and neck. Methods: A systematic review was conducted according to the Preferred Reporting Items for Systematic Reviews and Meta-Analyses guidelines. We searched the PubMed, Embase, Cochrane Library, and Web of Science databases between 7th and 10th November 2023. Results: A total of 2962 patients from 26 studies between 2007 and 2023 were included in this meta-analysis. The median RT dose was 64 Gy (range: 56–66 Gy). The median proportions of high-grade, pathological tumor stage 3 or 4 and pathological lymph node involvement were 42% (0–100%), 40% (0–77%), and 31% (0–75%). The pooled locoregional control rates at 3, 5, and 10 years were 92% (95% confidence interval [CI], 89–94%), 89% (95% CI, 86–93%), and 84% (95% CI, 73–92%), respectively. The pooled disease-free survival (DFS) rates at 3, 5, and 10 years were 77% (95% CI, 70–83%), 67% (95% CI, 60–74%), and 61% (95% CI, 55–67%), respectively. The pooled overall survival rates at 3, 5, and 10 years were 84% (95% CI, 79–88%), 75% (95% CI, 72–79%), and 68% (95% CI, 62–74%), respectively. Severe late toxicity ≥ grade 3 occurred in 7% (95% CI, 3–14%). Conclusion: PORT showed favorable long-term efficacy and safety in SGC, especially for patients with high-grade histology. Considering that DFS continued to decrease, further clinical trials exploring treatment intensification are warranted.

## 1. Introduction

Salivary gland cancer (SGC) is a rare malignancy that accounts for less than 5% of all head and neck cancers and encompasses a widely heterogeneous histology, with more than 20 different subtypes, according to the latest World Health Organization (WHO) classification [1]. The most common subtypes include mucoepidermoid carcinoma (MEC), acinic cell carcinoma, adenoid cystic carcinoma (ACC), carcinoma ex pleomorphic adenoma (Ca ex PA), and adenocarcinoma not otherwise specified [2]. In addition, SGC exhibits different rates of incidence and prevalence depending on the anatomical site [3]. Most cases occur in the parotid gland, followed by submandibular, sublingual, and minor salivary glands [4]. The probability of cancer in a parotid mass ranges from 15% to 32% compared to that from 41% to 50% in a submandibular mass, 70% to 90% in minor salivary gland masses, and almost 100% in sublingual masses [5]. Given the low incidence, heterogeneous behavior, and diverse anatomical sites of SGC, there are limited clinical studies on its management. Additionally, only a few guidelines have recently been published to provide relevant practical recommendations for patients with SGC [6,7,8,9,10].

Although there are no randomized trials comparing surgery alone with surgery followed by postoperative radiotherapy (PORT) in patients with SGC of the head and neck, several retrospective studies have shown the effectiveness of PORT in patients with adverse prognostic factors. The American Society of Clinical Oncology (ASCO) guidelines recommend that PORT should be offered to all patients with resected ACC and those with high-grade tumors, positive resection margin (RM), lymph node (LN) metastases, perineural invasion (PNI), lymphovascular invasion (LVI), or T3–4 tumors [6]. PORT may be offered to patients with close RM or intermediate-grade tumors. The European Society for Medical Oncology (ESMO)—European Reference Network on Rare Adult Solid Cancers (EURACAN) guidelines recommend PORT for patients with T3–4, high/intermediate-grade tumors, close and/or positive RM, and/or PNI [7]. The National Comprehensive Cancer Network (NCCN) guidelines recommend PORT as the preferred modality for patients with T3–4, LN metastases, high/intermediate-grade tumors, close or positive RM, PNI, and/or LVI [9]. However, these guidelines and consensus were mainly based on retrospective studies with a limited number and great heterogeneity among the study patients. To date, there are no meta-analyses about treatment outcomes encompassing the diverse and heterogeneous features of SGC treated with PORT.

Therefore, we conducted a systematic review and meta-analysis to report the cumulative evidence on PORT for SGC of the head and neck. 

## 2. Materials and Methods

This systematic review was conducted according to the Preferred Reporting Items for Systematic Reviews and Meta-Analyses guidelines [11]. Although prospective registration of systemic reviews is generally recommended, there is no information on the overall processing time in PROSPERO, and some state that it may take up to several months [12]. A recent study reported the median time from registering a protocol in PROSPERO to publication was 16 months [13]. This systematic review and meta-analysis was designed as an international cooperative research study and conducted on schedule without registration in PROSPERO.

### 2.1. Study Search

A literature search was conducted using the PubMed, Embase, Cochrane Library, and Web of Science databases between 7th and 10th November 2023. The keywords used regarding the patient/problem, intervention, comparison, and outcome (PICO) model are listed in Supplementary Table S1. We cooperated with a professional librarian at the Soonchunhyang University College of Medicine, Bucheon, to develop and review the search strategy. Studies on humans published in English from 1974 to 2023 were included. In addition, the reference lists of the review articles, relevant studies, and clinical practice guidelines were reviewed. A total of 5178 articles were identified, and three authors (J. Wang, X. Guo, and J. Yu) independently screened the article titles, abstracts, and full texts as necessary. Disagreements were resolved by a fourth author (S.H. Bae).

### 2.2. Selection Criteria

The inclusion criteria were as follows: (1) original studies including randomized controlled trials, nonrandomized clinical trials, case series, or observational studies on SGC of head and neck; (2) primary SGC diagnosed as per the WHO classification; (3) the inclusion of ≥10 patients who received surgery followed by PORT with curative intent; (4) the use of megavoltage equipment; (5) reporting of at least ≥2 years locoregional control (LRC) and/or survival and/or toxicities. In the absence of numerical data, the LRC and survival were assumed indirectly using descriptive plots. In cases of multiple studies from one institution with overlapping patients, the following criteria were applied to determine inclusion and were prioritized in numerical order: (1) studies that described treatment outcomes of SGC patients treated with PORT in detail, (2) studies with the largest number of patients, and (3) the most recently published study. Studies from the same institution were independently categorized if they were conducted during different periods. Additionally, the two treatment groups in one study were independently categorized if LRC and survival were reported separately. The exclusion criteria were as follows: (1) reviews, letters, comments, replies, editorials, and other nonoriginal studies, (2) duplicate patient data, (3) recurrent and/or metastatic SGC, (4) previous RT history for head and neck cancer, (5) intraoperative radiotherapy, (6) ^60^Co gamma ray, neutron therapy, brachytherapy, Gamma Knife, and charged-particle therapy. 

### 2.3. Data Extraction and Quality Assessment

Data extraction was carried out independently by four authors using a standardized form, and the following data were obtained: (1) study, patient, and tumor characteristics; (2) treatment; (3) survival; and (4) late toxicity. Survival rates at 3–10 years were investigated. For studies lacking reported survival rates while having available survival curves, the corresponding numeric rates were extracted from the survival curves by employing the Engauge Digitizer (version 12.1, http://markummitchell.github.io/engauge-digitizer. accessed on 5 December 2023). Late toxicities were defined according to the Common Terminology Criteria for Adverse Events or toxicity criteria of the Radiation Therapy Oncology Group/The European Organization for Research and Treatment of Cancer (RTOG/EOTRC). The overall incidence of late toxicities and severe toxicities ≥ grade 3 was assessed. 

Because most studies were retrospective, we used the Newcastle–Ottawa Scale (NOS) to assess study quality [14]. Studies with over 7 points were categorized as high-quality, and studies with scores 4–6 were categorized as medium-quality.

### 2.4. Statistical Analysis

The heterogeneity among studies was evaluated using the Higgins I^2^ statistic [15]. An I^2^ value > 50% corresponded to substantial heterogeneity. Given the variations in treatment decision-making and the periods for which the study was applicable, the random-effects model was considered superior to the fixed-effects model when calculating pooled estimates. The DerSimonian and Laird method was applied for the random-effects analysis, and we present both estimates in the tables [16]. Publication bias was assessed using funnel plots and Egger’s regression tests. If the funnel plot was symmetrical or the *p*-value exceeded 0.05 in Egger’s test, then the null hypothesis of no publication bias was accepted. For comparison between subgroups, a Q test based on an analysis of variance and a random effects model was used, and *p* < 0.05 was considered statistically significant. All statistical analyses were conducted using the Rex Excel-based statistical analysis software, version 3.6.0 (RexSoft, Seoul, Republic of Korea, http://rexsoft.org/).

**Table 1:** Study details for salivary gland carcinoma treated with surgery followed by postoperative radiotherapy.

Author	Country	Study Type	NOS	Time of Study	No.	Anatomical Site	Histology (%)	Grade H/I/L (%)	Surgery Type (%)	RT Technique	Median Total Dose (Gy) (Range)	RT Target	Post-op CCRT (%)
Yan, 2023 [17]	China	S/R	5	2004–2020	418	Major salivary glands	All subtypes	22/0/78	-	3D, IMRT	-	-	11
Park, 2023 [18]	Korea	M/R	6	2004–2019	118	Parotid gland	All subtypes	42/8/50	P (83)/P + LND (17)	3D, IMRT	63 (54–78.75)	TB (71)/TB + NI (29)	2
Duru Birgi, 2023 [19]	Turkey	S/R	6	2013–2018	18	Parotid gland	All subtypes	-	P (67)/ P + LND (33)	IMRT	66 (60–70)	TB (39)/TB + NI (61)	17
Hsieh_A, 2023 [20]	Taiwan	M/R	7	2000–2015	263	All salivary glands	All subtypes	72/28 ^a^	P ± LND	3D, IMRT	62 ± 9 ^b^	TB + NI	0
Hsieh_B, 2023 [20]	Taiwan	M/R	7	2000–2015	148	All salivary glands	All subtypes	88/12 ^a^	P ± LND	3D, IMRT	65 ± 8 ^b^	TB + NI	100
Zang, 2022 [21]	China	S/R	5	2009–2016	60	Major salivary glands	All subtypes	-	P (30)/ P + LND (70)	IMRT	63 (60–68)	TB (7)/TB + NI (93)	7
Franco, 2021 [22]	USA	S/R	5	2008–2020	72	All salivary glands	All subtypes excluding ACC	-	-	IMRT	-	-	42
Dou, 2019 [23]	China	S/P2	6	2016–2018	52	All salivary glands	Intermediate or high-grade histology	-	-	IMRT	NR (60–66)	-	100
Nutting_A, 2018 [24]	UK	M/P3	7	2008–2013	54	Parotid gland	All subtypes	43/17/30	-	3D	65 (58–65)	TB+/−NI	0
Nutting_B, 2018 [24]	UK	M/P3	7	2008–2013	56	Parotid gland	All subtypes	32/20/38	-	IMRT	65 (60–65)	TB+/−NI	0
Nishikado, 2018 [25]	Japan	S/R	4	1999–2007	58	Parotid gland	All subtypes	-	-	-	60 ^c^ (NR)	-	0
Li, 2018 [26]	China	M/P2	6	2013–2016	20	All salivary glands	Intermediate/high-grade histology	40/60/0	-	3D, IMRT	66 (NR)	TB + NI	100
Gebhardt, 2018 [27]	USA	S/R	6	2002–2015	128	All salivary glands	All subtypes	45/23/24	P (47)/ P + LND (53)	IMRT	66 (45–70.2)	TB (17)/TB + NI (83)	22
Boon, 2018 [28]	Netherland	M/R	5	2000–2016	15	All salivary glands	Secretory carcinoma with ETV6-NTRK3 fusion gene	0/0/100	P ± LND	-	66 (60–66)	TB (27)/TB + NI (27)	0
Zhang, 2017 [29]	China	S/R	5	2008–2014	30	Parotid gland	All subtypes	-	P (33)/ P + LND (67)	2D, IMRT	NR (60–70)	TB+/−NI	0
Gutschenritter, 2017 [30]	USA	S/R	4	2002–2014	78	All salivary glands	All subtypes	-	P ± LND	3D, IMRT	NR (50–66)	-	0
Sayan, 2016 [31]	USA	S/R	5	2006–2015	20	Major salivary glands	All subtypes	-	P (60)/ P + LND (40)	3D, IMRT	60 (NR)	TB (55)/TB + NI (45)	0
Mifsud_A, 2016 [32]	USA	S/R	6	1998–2013	103	All salivary glands	All subtypes	37/26/29	-	-	64 (45–72) ^d^	-	0
Mifsud_B, 2016 [32]	USA	S/R	6	1998–2013	37	All salivary glands	All subtypes	73/5/11	-	-	64 (45–72) ^d^	-	100
Hosni, 2016 [33]	Canada	S/R	7	2000–2012	304	Major salivary glands	All subtypes	41/21/38	P (49)/ P + LND (51)	3D, IMRT	66 (46–74)	TB (62)/TB + NI (38)	3
Haderlein, 2016 [34]	Germany	S/R	5	2000–2014	63	All salivary glands	All subtypes	64/19/14	P (6)/ P + LND (94)	3D, IMRT	64 (45–74)	TB (25)/TB + NI (75)	46
Kaur, 2014 [35]	India	S/R	5	1998–2008	39	Major salivary glands	All subtypes	-	P ± LND	2D, 3D	60 (24–64)	-	0
Tam, 2013 [36]	USA	S/R	6	1990–2011	200	Major salivary glands	All subtypes	-	P (64)/ P + LND (36)	2D, 3D, IMRT	63 (60–66)	TB+/−NI	10
Chung, 2013 [37]	USA	S/R	5	1998–2011	37	Major salivary glands	All subtypes excluding ACC	-	P (65)/ P + LND (35)	3D, IMRT	60 (46–70)	TB+/−NI	24
Kim, 2012 [38]	Korea	S/R	5	1998–2010	35	Major salivary glands	Salivary duct carcinoma	100/0/0	P (11)/P + LND (89)	-	59.4 (50.4–71.4)	TB + NI (100)	9
Al-Mamgani, 2012 [39]	Netherlands	S/R	6	1995–2010	186	Parotid gland	All subtypes	40/10/41	P (77)/ P + LND (23)	2D,3D, IMRT	66 (54–70)	TB+/−NI	2
Pederson, 2011 [40]	USA	M/R	5	1991–2007	24	All salivary glands	All subtypes	79/0/21	P (12)/ P + LND (88)	2D,3D, IMRT	65 (55–68)	-	100
Noh, 2010 [41]	Korea	S/R	5	1995–2006	75	Major salivary glands	All subtypes	83/0/17	P (64)/ P + LND (36)	3D	56 (54–70)	TB (73)/P + NI (27)	0
Chen, 2007 [42]	USA	S/R	7	1960–2004	251	All salivary glands	All subtypes	-	-	2D,3D, IMRT	63 (45–72)	TB (48)/TB + NI (52)	4

S, single center; M, multicenter; R, retrospective study; P2, prospective phase 2 study; P3, prospective phase 3 study; NOS, the Newcastle–Ottawa Scale; No., number of patients; NR, not reported; ACC, adenoid cystic carcinoma; H, high-grade; I, intermediate-grade; L, low-grade; P, primary resection; LND, lymph node dissection; 2D, two-dimensional radiotherapy; 3D, three-dimensional conformal radiotherapy; IMRT, intensity-modulated radiotherapy; TB, tumor bed; NI, nodal irradiation; postop CCRT, postoperative concurrent chemoradiotherapy. ^a^ means patients with a high grade versus patients with an intermediate/low grade. ^b^ means mean dose ± standard deviation. ^c^ means mean dose. ^d^ means the median dose which the entire patients received.

## 3. Results

### 3.1. Search Result

**Figure 1:**
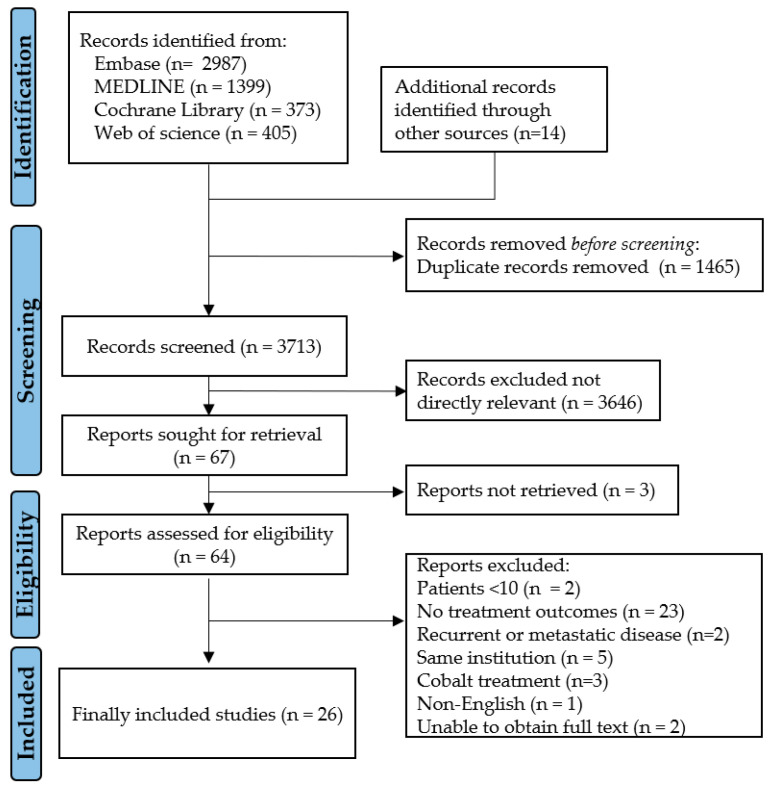
PRISMA (Preferred Reporting Items for Systematic Reviews and Meta-Analyses) algorithm.

A total of 5164 studies were initially screened from the four databases, and 14 additional studies were added through cross-referencing. After excluding 1465 duplicate studies, the remaining 3713 studies were identified and screened. After screening the titles and abstracts, 64 studies were selected for full-text reviews. Finally, a total of 2962 patients from 26 studies were selected for this systematic review and meta-analysis, as shown in Figure 1 [17,18,19,20,21,22,23,24,25,26,27,28,29,30,31,32,33,34,35,36,37,38,39,40,41,42]. Among these, three studies separately analyzed the outcomes of patients who received PORT in two treatment groups, and each treatment group was categorized into a different cohort [20,24,32]. Therefore, a total of 29 cohorts were included in this study.

### 3.2. Selected Studies’ Characteristics

Table 1 and Table 2 present the characteristics of the 29 cohorts in 26 studies conducted between 2007 and 2023. Three studies were prospective, and the remaining were retrospective. The quality of each study according to the NOS is presented in Table 1.

Six studies included only carcinomas in the parotid gland, followed by carcinomas in the major salivary glands (n = 9), and carcinomas in all salivary glands (n = 11). Most studies included all the histological subtypes. The remaining six studies included only specific histological subtypes: intermediate- to high-grade subtypes (n = 2), all subtypes excluding ACC (n = 2), secretory carcinoma with the ETV6-NTRK3 fusion gene (n = 1), and salivary duct carcinoma (n = 1). The proportion of high-grade histology was 0–100% (median, 42%). Individual anatomical sites and histological data are summarized in Supplementary Table S2. Facial nerve palsy or dysfunction as the initial symptom was present in 3–8% [18,21,38]. Lymph node dissection (LND) was conducted in 17–94% (median, 46%). Pathological tumor (pT) stage 3 or 4 and pathological LN (pN) involvement were 0–77% (median, 40%), and 0–75% (median, 31%). LVI and PNI were detected in 0–51% (median, 20%) and 10–84% (median, 39%). The definition of close and positive RMs was variable according to the study (Supplementary Table S2), and positive RM was 4–93% (median, 46%). RT was delivered using two-dimensional (2D) RT, three-dimensional conformal RT (3DCRT), and intensity-modulated RT (IMRT). The median RT dose was 64 Gy (range: 56–66 Gy). The proportion of nodal irradiation, including the involved LN and/or elective LN regions, was described in only 11 studies and ranged from 27% to 100% (median, 52%). Concurrent chemoradiotherapy (CCRT) was applied in 0–100% (median, 4%).

### 3.3. Survivals

The median follow-up period was 50 months (range: 11–131 months). The median 3-, 5-, and 10-year LRC rates were 91% (range: 77–99%), 89% (range: 63–97%), and 82% (range: 63–96%), respectively. The median 3- and 5-year distant metastasis-free survival (DMFS) rates were 83% (range, 53–95%) and 75% (range: 62–80%), respectively. Accordingly, the 3- and 5-year disease-free survival (DFS) rates were 79% (range: 42–100%) and 61% (range: 27–89%). The median 3-, 5-, and 10-year overall survival (OS) rates were 85% (range: 52–100%), 77% (range: 55–89%), and 68% (range: 57–89%), respectively (Table 2). Using the random effects model, the pooled 3-, 5-, and 10-year LRC rates were 92% (95% confidence interval [CI], 89–94%), 89% (95% CI, 86–93%), and 84% (95% CI, 73–92%), respectively. The pooled 3- and 5-year DMFS rates were 81% (95% CI, 76–86%) and 74% (95% CI, 70–79%), respectively, whereas the pooled 3- and 5-year DFS rates were 77% (95% CI, 70–83%) and 67% (95% CI, 60–74%), respectively. The pooled 3-, 5-, and 10-year OS rates were 84% (95% CI, 79–88%), 75% (95% CI, 72–79%), and 68% (95% CI, 62–74%), respectively.

**Figure 2:**
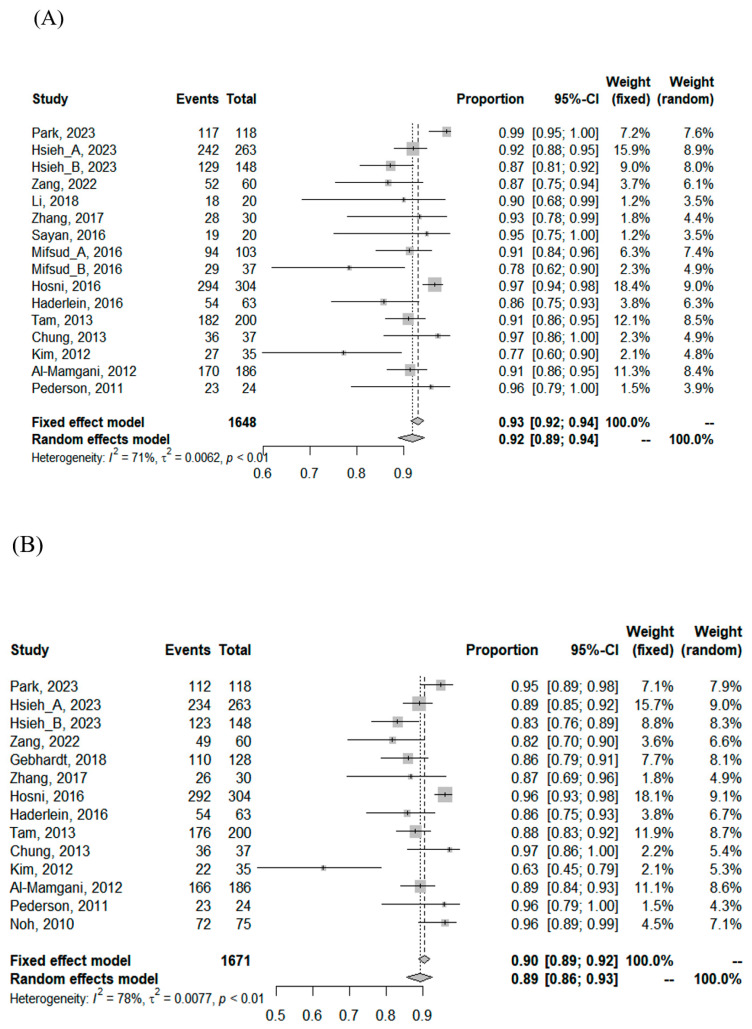

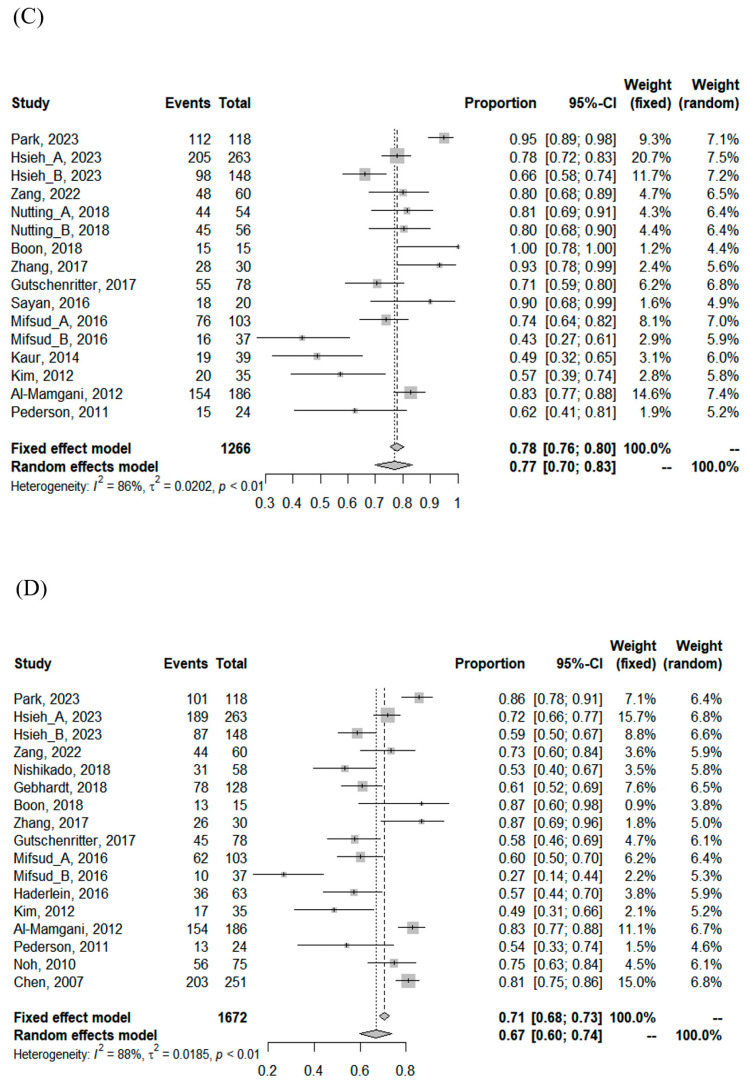

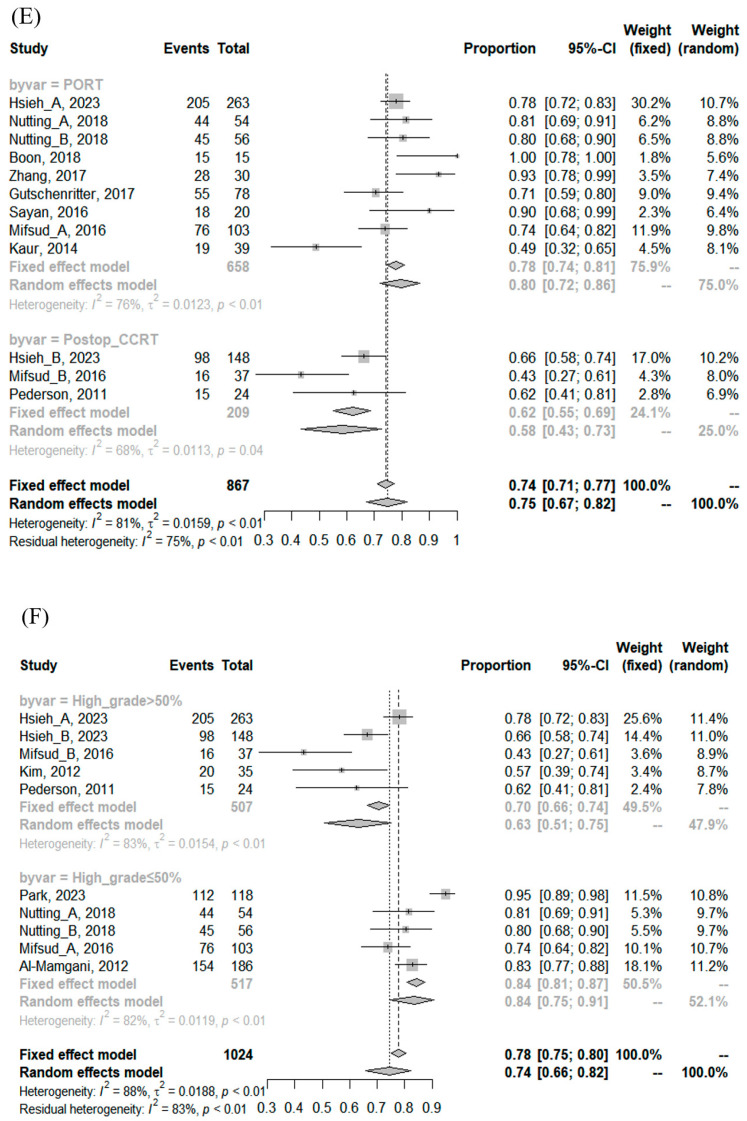
Forest plot of (**A**) 3-year locoregional control (LRC), (**B**) 5-year LRC, (**C**) 3-year disease-free survival (DFS), (**D**) 5-year DFS, (**E**) 3-year DFS between cohorts treated with postoperative concurrent chemoradiotherapy (Post-op_CCRT) versus cohorts with postoperative radiotherapy alone (PORT), and (**F**) 3-year DFS between cohorts with the proportion of a high grade ≥ 50% versus cohorts with the proportion of a high grade < 50%.

There was significant heterogeneity among the included cohorts for survival estimates (Table 3); however, publication bias was not detected (Supplementary Figure S1). In the subgroup comparison, cohorts with the proportion of a high grade < 50% had significantly better 3- and 10-year LRC, 3-year DFS, and 3- and 5-year DMFS than cohorts with the proportion of a high grade ≥ 50%. Cohorts treated with postoperative CCRT had significantly inferior 3-year DFS and 10-year OS than cohorts treated with PORT alone. No association was found between the RT dose and any survival index, the details of which are summarized in Supplementary Table S3 and Figure 2.

### 3.4. Late Toxicities

The overall incidence and evaluated types of late toxicities were variable among the included studies, as shown in Table 4. The pooled rates assessed using the random-effects model for the overall incidences of xerostomia, hearing impairment, and osteoradionecrosis were 37% (95% CI, 11–68%), 25% (95% CI, 7–49%), and 1% (95% CI, 1–2%), respectively. Severe late toxicity ≥ grade 3 occurred in 0–34%, and the pooled rate was 7% (95% CI, 3–14%), respectively (Supplementary Figure S2).

## 4. Discussion

The principal treatment for SGC is surgical resection with adequate free margins. PORT is considered for patients with adverse prognostic factors; however, its survival benefit is unclear. Gutschenritter et al. [30] reported no statistically significant difference in survival between patients who underwent surgery alone and those who underwent surgery followed by PORT for SGC: 72% vs. 58% at 5-year DFS rates; and 88% vs. 68% at 5-year OS rates, respectively. Noh et al. [41] showed a similar 5-year LCR between surgery alone and PORT (100% vs. 96%) for major SGC, despite PORT being administered only to patients with high-risk factors. However, the 5-year DFS and OS rates were lower in the PORT (74% and 78%, respectively) than in the surgery-alone (95% and 100%, respectively) group. A recent study on parotid gland cancer reported that PORT was associated with a significant improvement in the 5-year LRC (*p* = 0.005) and DFS (*p* = 0.009) compared with surgery alone [18]. To overcome the limitations of these retrospective studies with a small number of patients and an imbalance in prognostic factors between the treatment groups, several studies using the National Cancer Database (NCDB) have been published. A study of 4068 patients with SGC with pT1–4NX–1M0 high-grade tumors, pT3–4NX–0M0, or pT1–4N1M0 low-grade tumors showed a statistically improved OS with PORT, with minimal absolute benefit (56% vs. 51% at 5 years) [43]. Another two studies (4145 patients with SGC who were treated with primary resection and LND [44] and 7342 patients with SGC who had MEC, acinic cell carcinoma, ACC, adenocarcinoma, or Ca ex PA [45]) also showed better OS with PORT. Collectively, the three NCDB studies enrolled almost all contemporary patient cohorts treated between 2004 and the early 2010s.

To the best of our knowledge, the current study is the first systematic review and meta-analysis focusing on the treatment outcomes of patients with SGC who underwent surgery followed by PORT. Compared with the abovementioned studies, our pooled cohort included more recent patients who were treated up to 2020, implying a higher proportion of more advanced RT techniques and a more meticulous histological classification being applied. The pooled 5-year LRC, DFS, and OS rates were 89% (95% CI, 86–93%), 67% (95% CI, 60–74%), and 75% (95% CI, 72–79%), respectively. The favorable survival compared to that reported in previously published studies supports the efficacy of PORT as a local modality. Considering that the LRC, DMFS, and DFS continue to decrease, however, further clinical trials are warranted to improve both locoregional and distant tumor control.

In the subgroup analysis, cohorts with a proportion of a high grade ≥ 50% had statistically worse 3- and 10-year LRC, 3-year DFS, and 3- and 5-year DMFS. A high grade is one of the most important risk factors for the recurrence of SGC, and all guidelines recommend PORT for patients with high-grade tumors [6,7,8,9]. On the other hand, the necessity of PORT is controversial in cases of low- and intermediate-grade tumors. One NCDB study of 744 patients with intermediate-grade, early T-stage, LN-negative parotid cancer reported that PORT significantly and independently improved survival only in patients with positive RM [46]. A Canadian-led multicenter retrospective study of 621 patients with low- or intermediate-grade major SGC estimated that the marginal probability of locoregional recurrence (LRR) within 10 years was 15.4% without PORT and 8.8% with PORT on the multivariable model [47]. The authors suggested that PORT may reduce LRR in some patients with low- and intermediate-grade SGC with advanced pT stage, LVI, and RM (+). The study, using the Taiwan Cancer Registry and National Health Insurance Research Database, analyzed 655 patients with early-stage major SGC [48]. No significant differences were noted in the LRR and disease-specific survival between patients who received PORT and those who did not. Although RM (+) patients had a higher LRR, the stratified analysis indicated that the use of PORT had no protective effects. The status of RM is generally considered a major determinant in applying PORT for low- and intermediate-grade SGC; however, the definition of close and positive RM varies among studies, as shown in Supplementary Table S2. This discrepancy might have caused the conflicting results of PORT in terms of survival. Subgroup analysis on RM in the current meta-analysis was challenging because of the lack of individual patient data and variable definitions of RM. Efforts should be made to establish a uniform definition of the proper RM for SGC and assess whether the status of RM truly affects survival in low-to intermediate-grade SGC.

Compared with good LCR achieved by surgery followed by PORT, suboptimal survival and DM-dominant recurrent patterns of SGC require the development of several intensified treatment strategies, such as postoperative CCRT. Notwithstanding, this meta-analysis showed that postoperative CCRT led to significantly inferior 3-year DFS and 10-year OS rates to those of PORT. Among the 26 studies, three retrospective studies compared PORT with postoperative CCRT, and the cohorts receiving postoperative CCRT apparently harbored more adverse prognostic factors than those receiving PORT, with statistical significance [20,31,32]. Overall, no statistically significant survival benefit existed from postoperative CCRT, with only one study showing improved long-term OS and PFS in SGC patients with LN metastases and superior LRC in patients with R2 resection or ACC [20]. The NCDB studies failed to show improved outcomes with the addition of chemotherapy to PORT in SGC patients [49,50]. Another NCDB study reported that postoperative CCRT was associated with increased mortality on both multivariable and propensity score-adjusted analyses (hazard ratio [HR]:1.39; 95% CI: 1.07–1.79 and HR: 1.49; 95% CI: 1.14–1.94, respectively) [51]. Therefore, the current level of evidence on postoperative CCRT is still low in unselected patients with SGC, and this treatment strategy is not recommended outside clinical studies. The ongoing phase III RTOG 1008 study (NCT01220583), which compares PORT with postoperative CCRT using weekly cisplatin in patients with high-risk SGC after surgery, might provide some answers.

This meta-analysis includes studies that used megavoltage equipment and reflects the treatment outcomes of modern RT techniques for SGC. Because the theoretical advantages of IMRT dose distributions over 2DRT and 3DCRT are generally accepted, IMRT has been routinely used throughout all tumor sites, and guidelines for SGC also recommend the use of IMRT [6,7,52,53]. In the current meta-analysis, the pooled rate for severe toxicity ≥ grade 3 was 7% (95% CI, 3–14%), indicating that PORT can be used as a local modality with an acceptable level of safety. Although IMRT has changed the practice of RT, it is unclear whether its use provides a clinically relevant advantage in SGC [54]. The overall incidence of late toxicity varied among the included cohorts, as presented in Table 4. Among these, two studies focused on RT-related toxicity in SGC. A phase 3 trial comparing 3DCRT with cochlear-sparing IMRT for parotid gland cancer showed no significant differences in hearing loss or other secondary endpoints, including patient-reported hearing outcomes, although the median dose to the ipsilateral cochlea was significantly reduced (56 Gy with 3DCRT vs. 36 Gy with IMRT, *p* < 0.0001) [24]. The second was a retrospective study that evaluated trigeminal nerve toxicity after IMRT for resected parotid gland cancer [19]. Grades 1 and 2 of trigeminal nerve toxicities occurred in 22% and 39% of patients, respectively, which were higher than the incidence of cranial nerve toxicity of 4–31% observed in head and neck studies treated with definitive RT [55,56,57]. The authors suggested that surgical interventions could potentially induce alterations in the postoperative tissues, leading to increased susceptibility. Therefore, further observations are required to evaluate the long-term safety of IMRT for SGC.

This study has some limitations. First, prospective or retrospective studies were included, except for one phase 3 study that focused on RT-related toxicity. The heterogeneity of observational studies and selection bias may have affected the pooled analysis [58]. In addition, there was a time interval between the literature search and publication, and this might give rise to publication bias. Considering that the median time from the literature search to publication was 8 months, and the time interval of recent meta-analyses was not significantly different, our systematic review provides timely, up-to-date evidence [13,59]. Second, 26 studies published between 2007 and 2023 were included in this analysis. The WHO classification system for SGC was updated until 2022, and the classification of histological subtypes changed several times during this period. Boon et al. [28] found that secretory carcinoma characterized by the ETV6-NTRK3 fusion gene, a new subtype of SGC in 2010, was previously diagnosed as acinic cell carcinoma, polymorphous adenocarcinoma, or adenocarcinoma not otherwise specified. However, this effect may have been minimized, because the current study included all the histological subtypes. Lastly, we included the use of megavoltage equipment and excluded studies treated with ^60^Co gamma rays. In addition, we did not permit duplicate patient data and selected the most recent study from one institution. This might reduce the number of late toxicities, and long-term outcomes and toxicities were not complete in some studies. Among the twenty-six studies, nine studies reported 10-year survival outcomes, and eleven evaluated late treatment-related toxicities. Further studies are needed to validate the long-term efficacy and safety of PORT for SGC.

## 5. Conclusions

The current systematic review and meta-analysis comprehensively demonstrated the short- and long-term survival and toxicities of PORT as a local treatment modality for SGC. High-grade histology has been confirmed to be a strong indicator of the utilization of PORT, whereas the value of PORT in low- to intermediate-grade tumors is still questionable and needs further assessment. Considering the suboptimal DFS and DM-dominant recurrent patterns, an intensified treatment strategy would be needed. However, concurrent chemotherapy accompanied by PORT may not be a good option as an intensified modality based on the pooled data. Further prospective investigations are warranted.

## Figures and Tables

**Table 2:** Pathologic details and treatment outcomes for salivary gland carcinoma treated with surgery followed by postoperative radiotherapy.

Author	pTstage: 1/2/3/4(%)	pNstage: 0/1/2/3(%)	LVI (%)	PNI (%)	Median f/u (mo)	3-yr LRC (%)	5-yr LRC (%)	10-yr LRC (%)	3-yr DMFS (%)	5-yr DMFS (%)	10-yr DMFS (%)	3-yr DFS (%)	5-yr DFS (%)	10-yr DFS (%)	3-yr OS (%)	5-yr OS (%)	10-yr OS (%)
Yan [17]	28/48/11/13	67/13/17/3	8	26	60	-	-	-	79	72	59	-	-	-	90	81	63
Park [18]	30/30/32/8	100/0/0/0	16	20	-	99	95	-	-	-	-	95	86	-	-	-	-
Duru Birgi [19]	22/45/11/0	56/11/11/0	0	11	30	-	-	-	-	-	-	-	-	-	-	-	-
Hsieh_A [20]	30/33/19/18	85/3/12/0	13	39	131 ^c^	92	89	85	-	-	-	78	72	64	88	80	72
Hsieh_B [20]	13/36/24/27	58/9/33/0	20	49	131 ^c^	87	83	82	-	-	-	66	59	53	80	71	61
Zang [21]	23/7 ^a^	68/13/19/0	-	32	56	87	82	82	85	78	66	80	73	63	91	85	85
Franco [22]	-	-	-	-	41	-	-	-	-	-	-	-	-	-	-	-	-
Dou [23]	-	-	-	-	16	-	-	-	-	-	-	-	-	-	-	-	-
Nutting_A [24]	30/28/13/22	59/7/26/0	-	-	50 ^c^	-	-	-	-	-	-	81	-	-	81	-	-
Nutting_B [24]	29/39/14/16	66/13/16/0	-	-	50 ^c^	-	-	-	-	-	-	81	-	-	86	-	-
Nishikado [25]	-	-	-	-	-	-	-	-	-	-	-	-	53	-	-	-	-
Li [26]	-	-	-	10	21	88	-	-	95	-	-	-	-	-	-	-	-
Gebhardt [27]	31/31/16/22	70/5/25/0	37	53	54	-	86	-	-	77	-	-	61	-	-	73.7	-
Boon [28]	47/47/0/0	93/0/7/0	-	-	-	-	-	-	-	-	-	100	89	89	100	89	89
Zhang [29]	-	-	-	-	-	92 ^d^	87 ^d^	-	-	-	-	93	88	-	87	82	-
Gutschenritter [30]	-	-	-	-	-	-	-	-	-	-	-	70	58	-	79	68	-
Sayan [31]	35/25/40/0	90/10/0/0	-	25	37	96	-	-	-	-	-	90	-	-	100	-	-
Mifsud_A [32]	32/23/23/22	80/6/14/0	21	53	35 ^c^	91	-	-	83	-	-	74	60	-	78	-	-
Mifsud_B [32]	16/14/30/40	35/14/51/0	38	84	35 ^c^	79	-	-	53	-	-	42	27	-	52	-	-
Hosni [33]	63/37 ^a^	73/9/18/0	22	53	82	97 ^d^	96 ^d^	96 ^d^	84	80	77	-	-	-	84	78	75
Haderlein [34]	24/15/44/14	49/22/29/0	-	60	31	86	86	-	-	62	-	-	58	-	-	63	-
Kaur [35]	-	-	-	-	11	-	-	-	-	-	-	49	-	-	-	-	-
Tam [36]	31/33/19/15	65/14/18/0	-	-	50	91 ^d^	88 ^d^	-	81	73	-	-	-	-	85	77	59
Chung [37]	14/30/16/40	51/49 ^b^	-	-	56	97	97	-	-	-	-	-	-	-	77	76	-
Kim [38]	17/31/43/9	26/74 ^b^	51	34	43	77	63	63	-	-	-	56	47	47	-	55	-
Al-Mamgani [39]	27/49/16/8	80/6/13/1	-	-	58	92	89	-	-	-	-	83	83	-	72	68	-
Pederson [40]	4/34/25/37	25/13/62/0	-	54	42	96	96	-	-	-	-	62	55	-	79	59	-
Noh [41]	21/28/35/16	81/7/12/0	16	19	-	-	96	-	-	-	-	-	74	-	-	78	-
Chen [42]	17/33/27/24	-	-	74	62	-	-	-	-	-	-	-	81	63	96	81	57

pTstage, pathologic tumor stage; pNstage, pathologic lymph node stage; LVI, lymphovascular invasion; PNI, perineural invasion; f/u, follow-up; mo, months; LRC, locoregional control; DMFS, distant metastases-free survival; DFS, disease-free survival; OS, overall survival. ^a^ means patients with pT1/2 versus patients with pT3/4. ^b^ means patients with pN0 versus patients with pN+. ^c^ means the median follow-up period of the entire patients. ^d^ reported local control rates.

**Table 3:** Pooled rates of survival.

Group	Cohorts (n)	Patients (n)	*p*, Heterogeneity	I^2^	Egger’s Test, *p*	Fixed Event Rate (95% CI)	Random Event Rate (95% CI)	*p* (between Groups)
3-year LRC								
All	16	1648	<0.0001	71.09%	0.1752	0.93 (0.92–0.94)	0.92 (0.89–0.94)	
Post-op CCRT ^a^	4	229	0.2824	21.32%	0.8796	0.88 (0.83–0.92)	0.88 (0.82–0.93)	0.0875
PORT ^b^	4	416	0.9845	0%	0.7215	0.93 (0.90–0.95)	0.93 (0.90–0.95)	
High grade > 50% ^c^	6	570	0.0313	59.25%	0.2016	0.89 (0.86–0.92)	0.87 (0.82–0.92)	0.0150
High grade ≤ 50%	5	731	0.0025	75.63%	0.5867	0.96 (0.94–0.97)	0.95 (0.91–0.98)	
mRT dose > 64 Gy	5	682	0.0035	74.52%	0.5371	0.94 (0.92–0.96)	0.93 (0.88–0.97)	0.5628
mRT dose ≤ 64 Gy	10	936	<0.0001	73.85%	0.3466	0.92 (0.90–0.94)	0.91 (0.87–0.95)	
5-year LRC								
All	14	1671	<0.0001	77.99%	0.3034	0.90 (0.89–0.92)	0.89 (0.86–0.93)	
Post-op CCRT	2	172	0.0943	64.28%	-	0.86 (0.80–0.91)	0.89 (0.75–0.98)	0.6259
PORT	3	368	0.1140	53.95%	0.8597	0.91 (0.88–0.94)	0.91 (0.85–0.96)	
High grade > 50%	6	608	0.0003	78.75%	0.7549	0.88 (0.85–0.90)	0.87 (0.80–0.93)	0.1827
High grade ≤ 50%	4	736	0.0009	81.73%	0.3854	0.93 (0.91–0.95)	0.92 (0.87–0.96)	
mRT dose > 64 Gy	5	790	<0.0001	84.57%	0.5832	0.91 (0.89–0.93)	0.90 (0.84–0.95)	0.7043
mRT dose ≤ 64 Gy	8	851	<0.0001	77.55%	0.6316	0.90 (0.87–0.92)	0.89 (0.84–0.93)	
10-year LRC								
All	5	810	<0.0001	91.93%	0.1546	0.89 (0.86–0.91)	0.84 (0.73–0.92)	
Post-op CCRT	1	148	-	-	-	0.82 (0.75–0.88)	0.82 (0.75–0.88)	0.3608
PORT	1	263	-	-	-	0.85 (0.81–0.89)	0.85 (0.81–0.89)	
High grade > 50%	3	446	0.0130	76.96%	0.0151	0.83 (0.79–0.86)	0.80 (0.70–0.88)	<0.0001
High grade ≤ 50%	1	304	-	-	-	0.96 (0.94–0.98)	0.96 (0.94–0.98)	
mRT dose > 64 Gy	2	452	<0.0001	95.66%	-	0.92 (0.90–0.95)	0.90 (0.72–0.99)	0.2421
mRT dose ≤ 64 Gy	3	358	0.0131	76.92%	0.3025	0.83 (0.79–0.87)	0.79 (0.66–0.89)	
3-year DFS								
All	16	1266	<0.0001	85.97%	0.6650	0.78 (0.76–0.80)	0.77 (0.70–0.83)	
Post-op CCRT	3	209	0.0421	68.44%	0.5343	0.62 (0.55–0.69)	0.58 (0.43–0.73)	0.0090
PORT	9	658	<0.0001	76.08%	0.4283	0.78 (0.74–0.81)	0.80 (0.72–0.86)	
High grade > 50%	5	507	<0.0001	83.26%	0.0811	0.70 (0.66–0.74)	0.63 (0.51–0.75)	0.0054
High grade ≤ 50%	5	517	0.0001	82.42%	0.7517	0.84 (0.81–0.87)	0.84 (0.75–0.91)	
mRT dose > 64 Gy	6	483	0.0002	79.55%	0.6255	0.78 (0.74–0.82)	0.80 (0.70–0.88)	0.4020
mRT dose ≤ 64 Gy	8	675	<0.0001	90.67%	0.3045	0.78 (0.75–0.81)	0.73 (0.60–0.84)	
5-year DFS								
All	17	1672	<0.0001	87.62%	0.1034	0.71 (0.68–0.73)	0.67 (0.60–0.74)	
Post-op CCRT	3	209	0.0022	83.69%	0.5542	0.53 (0.46–0.59)	0.47 (0.27–0.67)	0.0522
PORT	7	622	0.0008	73.74%	0.8001	0.68 (0.65–0.72)	0.69 (0.61–0.77)	
High grade > 50%	7	645	<0.0001	84.82%	0.0866	0.64 (0.60–0.67)	0.58 (0.46–0.68)	0.0780
High grade ≤ 50%	4	535	<0.0001	91.90%	0.4448	0.75 (0.71–0.78)	0.73 (0.59–0.85)	
mRT dose > 64 Gy	5	501	<0.0001	88.32%	0.8474	0.70 (0.66–0.74)	0.69 (0.55–0.81)	0.6700
mRT dose ≤ 64 Gy	10	1063	<0.0001	89.39%	0.0252	0.71 (0.68–0.74)	0.65 (0.55–0.74)	
10-year DFS								
All	6	772	0.0307	59.39%	0.8729	0.61 (0.58–0.65)	0.61 (0.55–0.67)	
Post-op CCRT	1	148	-	-	-	0.53 (0.45–0.61)	0.53 (0.45–0.61)	0.1010
PORT	2	278	0.0655	70.53%	-	0.66 (0.60–0.71)	0.73 (0.50–0.91)	
High grade > 50%	3	446	0.0379	69.44%	0.4465	0.59 (0.54–0.64)	0.57 (0.47–0.66)	-
High grade ≤ 50%	0	0	-	-	-	-		
mRT dose > 64 Gy	2	163	0.0080	85.78%	-	0.56 (0.48–0.64)	0.69 (0.34–0.95)	0.7471
mRT dose ≤ 64 Gy	4	609	0.3888	0.61%	0.2291	0.63 (0.59–0.67)	0.63 (0.59–0.67)	
3-year OS								
All	18	2284	<0.0001	84.23%	0.8229	0.84 (0.83–0.86)	0.84 (0.79–0.88)	
Post-op CCRT	3	209	0.0038	82.04%	0.6017	0.75 (0.69–0.81)	0.71 (0.52–0.87)	0.0755
PORT	8	619	0.0070	63.94%	0.4503	0.86 (0.83–0.89)	0.87 (0.81–0.92)	
High grade > 50%	4	472	<0.0001	87.60%	0.2295	0.83 (0.79–0.86)	0.77 (0.63–0.88)	0.5862
High grade ≤ 50%	6	1121	0.0481	55.24%	0.8811	0.80 (0.78–0.83)	0.80 (0.76–0.84)	
mRT dose > 64 Gy	7	787	0.0071	66.03%	0.4498	0.81 (0.78–0.84)	0.82 (0.76–0.87)	0.4812
mRT dose ≤ 64 Gy	8	971	<0.0001	89.66%	0.3286	0.88 (0.86–0.90)	0.85 (0.77–0.92)	
5-year OS								
All	17	2315	0.0001	64.47%	0.0875	0.77 (0.75–0.79)	0.75 (0.72–0.79)	
Post-op CCRT	2	172	0.2185	33.97%	-	0.70 (0.62–0.76)	0.68 (0.56–0.78)	0.0903
PORT	5	461	0.2404	27.17%	0.9974	0.79 (0.75–0.82)	0.78 (0.73–0.83)	
High grade > 50%	6	608	0.0021	73.40%	0.0424	0.74 (0.70–0.78)	0.70 (0.62–0.78)	0.2397
High grade ≤ 50%	4	1036	0.0030	78.45%	0.1743	0.77 (0.75–0.80)	0.76 (0.70–0.81)	
mRT dose > 64 Gy	6	805	0.0615	52.52%	0.6291	0.74 (0.70–0.77)	0.73 (0.68–0.78)	0.3252
mRT dose ≤ 64 Gy	8	984	0.0063	64.42%	0.1360	0.78 (0.75–0.81)	0.76 (0.71–0.81)	
10-year OS								
All	8	1659	<0.0001	85.03%	0.3694	0.67 (0.64–0.69)	0.68 (0.62–0.74)	
Post-op CCRT	1	148	-	-	-	0.61 (0.53–0.69)	0.61 (0.53–0.69)	0.0453
PORT	2	278	0.2472	25.32%	-	0.74 (0.68–0.79)	0.75 (0.65–0.85)	
High grade > 50%	2	411	0.0257	79.91%	-	0.68 (0.64–0.73)	0.67 (0.56–0.77)	0.8249
High grade ≤ 50%	2	722	0.0005	91.67%		0.68 (0.65–0.72)	0.69 (0.57–0.80)	
mRT dose > 64 Gy	3	467	0.0062	80.33%	0.9409	0.72 (0.67–0.76)	0.72 (0.60–0.83)	0.6456
mRT dose ≤ 64 Gy	4	774	<0.0001	89.58%	0.4103	0.65 (0.62–0.69)	0.68 (0.57–0.78)	

n, number; CI, confidence interval; LRC, locoregional control; post-op CCRT, postoperative concurrent chemoradiotherapy; PORT, postoperative radiotherapy; mRT dose, median radiotherapy dose; DFS, disease-free survival; OS, overall survival. ^a^ includes cohorts with all patients receiving post-op CCRT. ^b^ includes cohorts with all patients receiving PORT alone. Cohorts with mixed patients receiving post-op CCRT or PORT were excluded. ^c^ means that the proportion of patients with a high grade is greater than 50% among the entire patients.

**Table 4:** Late toxicities after surgery followed by postoperative radiotherapy for salivary gland cancer.

Author	Criteria	Overall Toxicity (%)	Severe Toxicity (%)
Park [18]	CTCAEv5.0	Hearing loss of Gr3 (1)	Gr3 (1)
Duru Birgi [19]	CTCAEv4.0	Trigeminal nerve toxicity of Gr1 (22) and Gr2 (39)	No ≥ Gr3 toxicity
Zang [21]	-	Xerostomia (30); Hearing impairment (28); Taste abnormalities (25); Paresthesia (23); Fibrosis of the skin (18); Trismus (10); ORN (3)	Gr3 (3)
Nutting_A [24]	CTCAEv3.0	Hearing toxicity of Gr1 (31), Gr2 (29), Gr3 (12), and Gr4 (2); OE of Gr1 (26), Gr2 (6), and Gr3 (2); OM of Gr1 (28), Gr2 (4), and Gr3 (2); Tinnitus of Gr2 (56) and Gr3 (2); Otalgia of Gr1 (24), Gr2 (10), and Gr3 (2); Skin pigmentation of Gr1 (49) and Gr 2 (6); Skin atrophy of Gr1 (48) and Gr2 (2); Skin fibrosis of Gr1 (52) and Gr2 (10); Functional mucous membrane toxicity of Gr1(20), Gr2 (8), and Gr3 (2); Clinical exam-mucous membrane toxicity of Gr1 (24) and Gr2 (4); Dry mouth of Gr1 (58), Gr2 (22), and Gr3 (4); Salivary gland changes of Gr1 (54) and Gr2 (20); ORN of Gr1 (2); Trismus of Gr1 (30) and Gr2 (6); Fatigue of Gr1 (26), Gr2 (10), and Gr3 (6)	Gr3 (32)/Gr4 (2)
Nutting_B [24]	CTCAEv3.0	Hearing toxicity of Gr1 (37), Gr2 (20), Gr3 (9), and Gr4 (7); OE of Gr1 (30) and Gr2 (7); OM of Gr1 (30) and Gr2 (4); Tinnitus of Gr1 (4), Gr2 (37), and Gr3 (7); Otalgia of Gr1 (28) and Gr2 (2); Skin pigmentation of Gr1 (43) and Gr 2 (4); Skin atrophy of Gr1 (44) and Gr2 (2); Skin fibrosis of Gr1 (56) and Gr2 (4); Functional mucous membrane toxicity of Gr1 (33) and Gr2 (9); Clinical exam-mucous membrane toxicity of Gr1 (24) and Gr2 (4); Dry mouth of Gr1 (72), Gr2 (20), and Gr3 (2)/Salivary gland changes of Gr1 (69), Gr2 (7), and Gr3 (2); ORN of Gr1 (2); Trismus of Gr1 (43) and Gr2 (6); Fatigue of Gr1 (40) and Gr2 (8)	Gr3 (20)/Gr4 (7)
Gebhardt [27]	CTCAEv4.0	Gr1 (52) and Gr2 (13)	No ≥ Gr3 toxicity
Sayan [31]	RTOG/EORTC	Xerostomia of Gr2 (25); Hearing loss (5)	Gr3 (5)
Hosni [33]	RTOG	ORN of Gr3 (1); Neck fibrosis of Gr3 (1); Trismus of Gr3 (0.3); Dysphagia of Gr3 (0.3)	Gr3 (3)
Tam [36]	CTCAEv4.0	Xerostomia of Gr 1–2 (54) and Gr 3 (1); Hearing loss of Gr 1–3 (19); PEG replacement (2); Radiation necrosis of Gr1 (1)	Gr3 (19)
Chung [37]	CTCAEv4.0	Hypothyroidism of Gr2, Xerostomia of Gr2, Trismus of Gr2; Fibrosis of Gr3 in 1 patient (3)	Gr3 (3)
Al-Mamgani [39]	CTCAEv3.0	Overall toxicities ≥ Gr2 (8); ORN of Gr3 (2); Hearing loss requiring hearing aid (6); Dysphagia and xerostomia of Gr2 (2); Subcutaneous toxicities of Gr2 (2)	Gr3 (9)
Pederson [40]	CTCAEv3.0	Xerostomia (21)/Esophageal stricture requiring dilatation (4)/TMJ syndrome (4)/Feeding tubes (13)	Gr3 (21)

CTCAE, the Common Terminology Criteria for Adverse Events; RTOG/EORTC, Toxicity criteria of the Radiation Therapy Oncology Group/The European Organization for Research and Treatment of Cancer; ORN, osteoradionecrosis; OE, otitis externa; OM, otitis media; TMJ, temporomandibular joint; Gr, grade.

## Data Availability

All data generated or analyzed during this study are included in this published article.

## Supplementary Materials

The following supporting information can be downloaded at: https://www.mdpi.com/article/10.3390/cancers16132375/s1, Table S1. Search strategy and results. Table S2. Tumor details for salivary gland carcinoma treated with surgery followed by postoperative radiotherapy. Table S3. Pooled rates of distant metastases free survival (DMFS). Figure S1. Funnel plots for survivals. Figure S2. Forrest plots of toxicities
