# Supplementary material for: The Current Position of Postoperative Radiotherapy for Salivary Gland Cancer: A Systematic Review and Meta-Analysis
Source: Cancers (Basel). 2024 Jun 28;16(13):2375. doi: 10.3390/cancers16132375 (PMC11240508; doi:10.3390/cancers16132375)
Supplement: Supplementary file 1 [file cancers-16-02375-s001.zip › 20240519 supplementary Figure S1.pptx]

## Slide 1
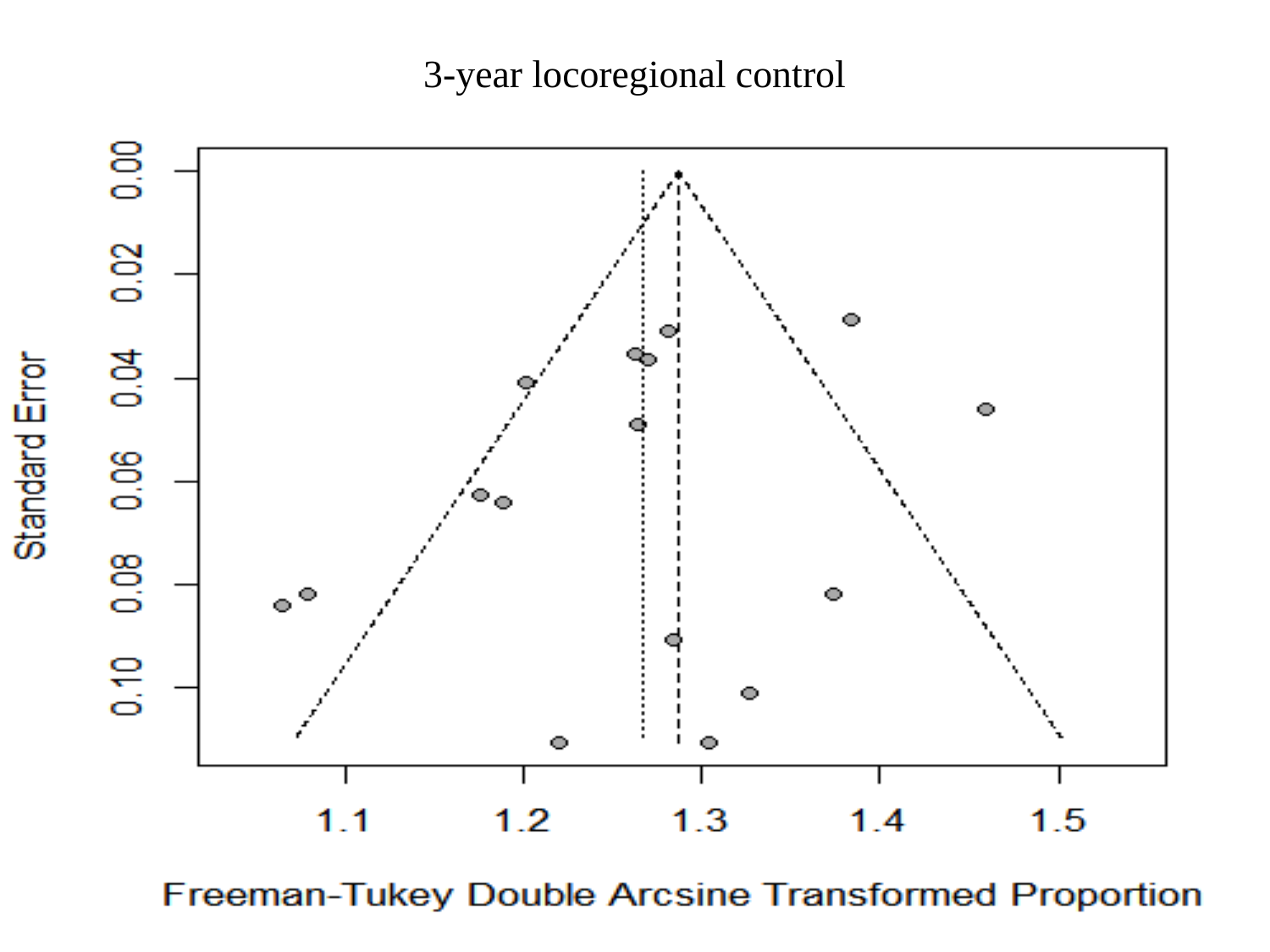

3-year locoregional control

## Slide 2
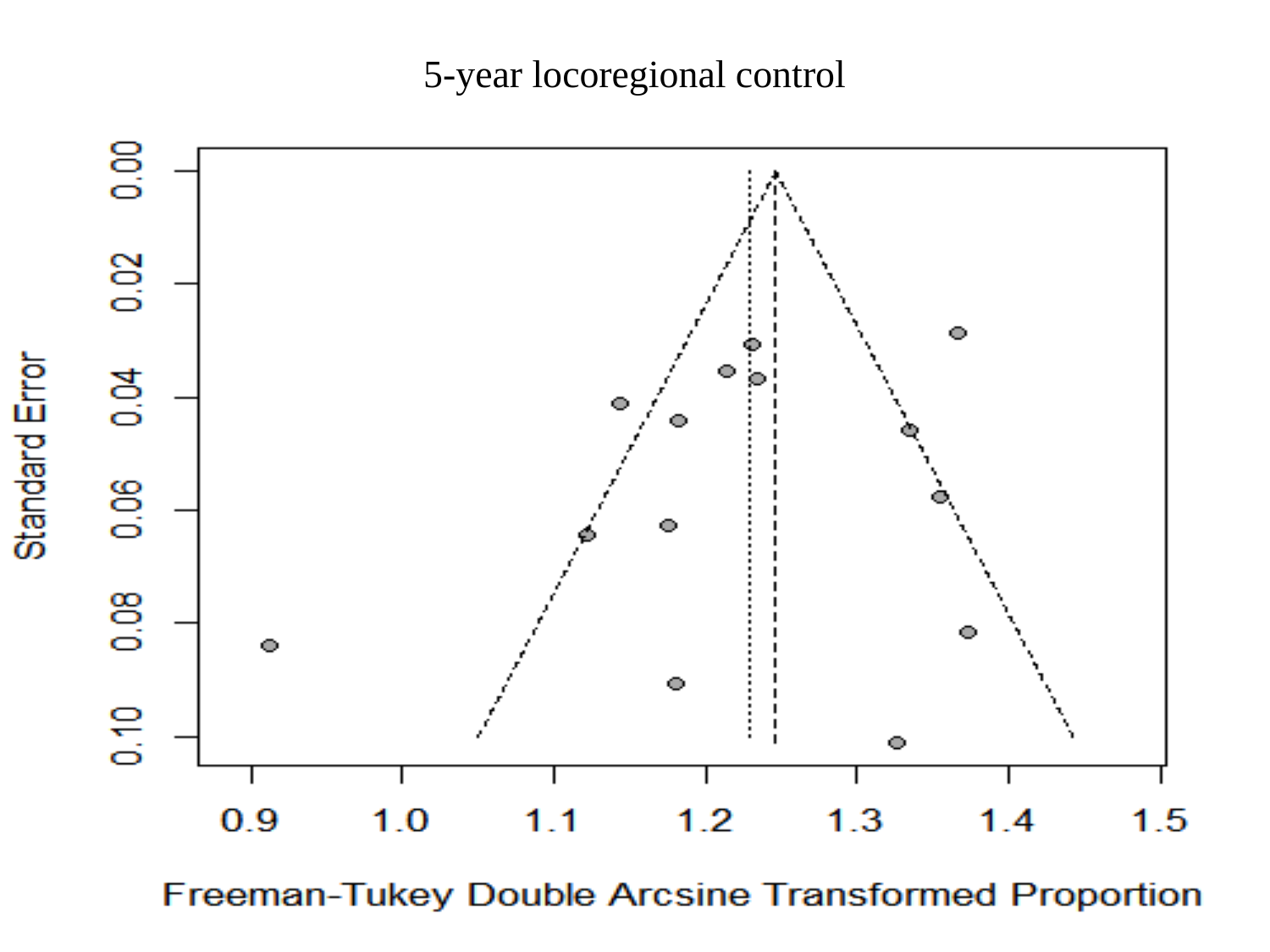

5-year locoregional control

## Slide 3
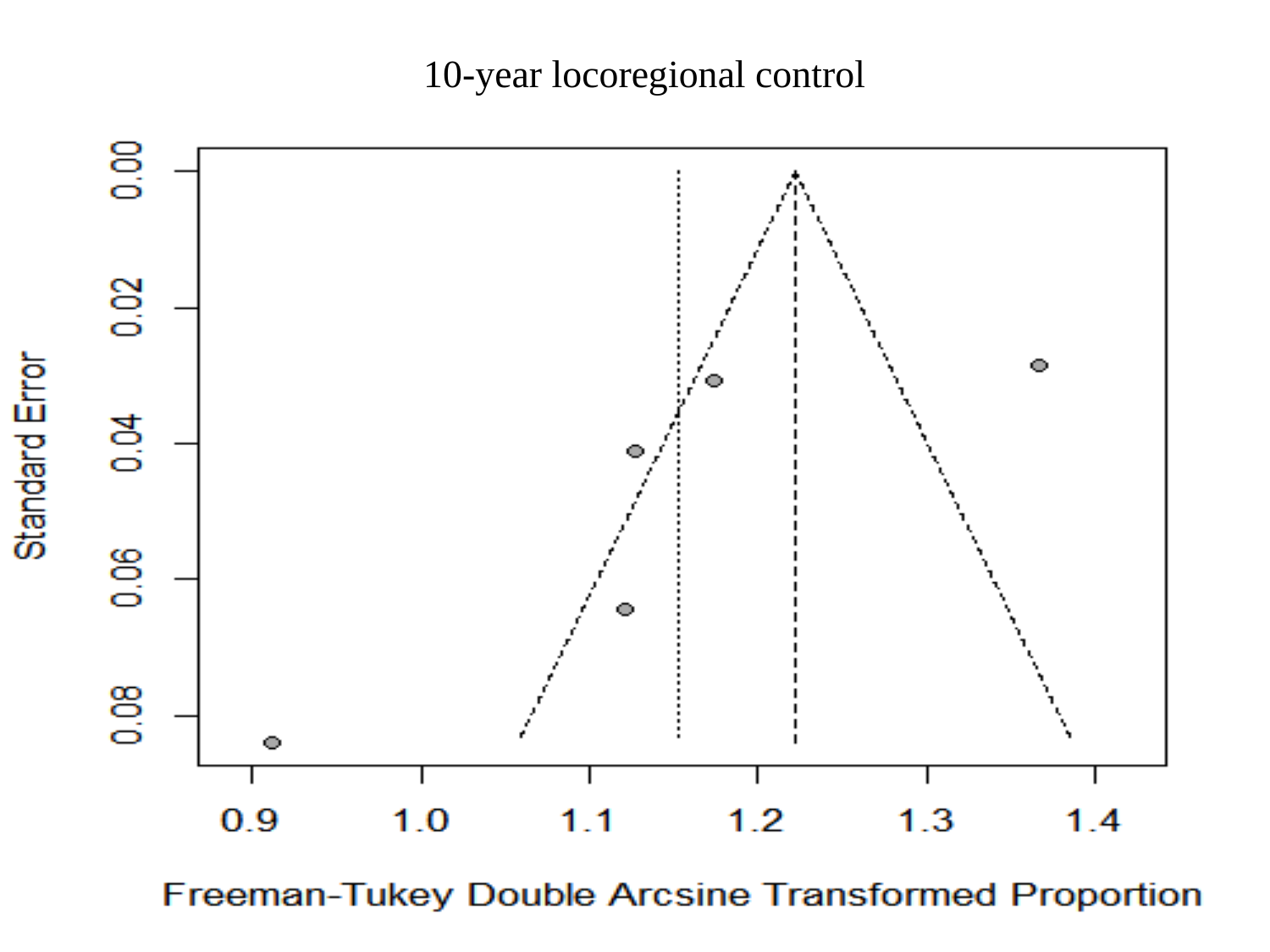

10-year locoregional control

## Slide 4
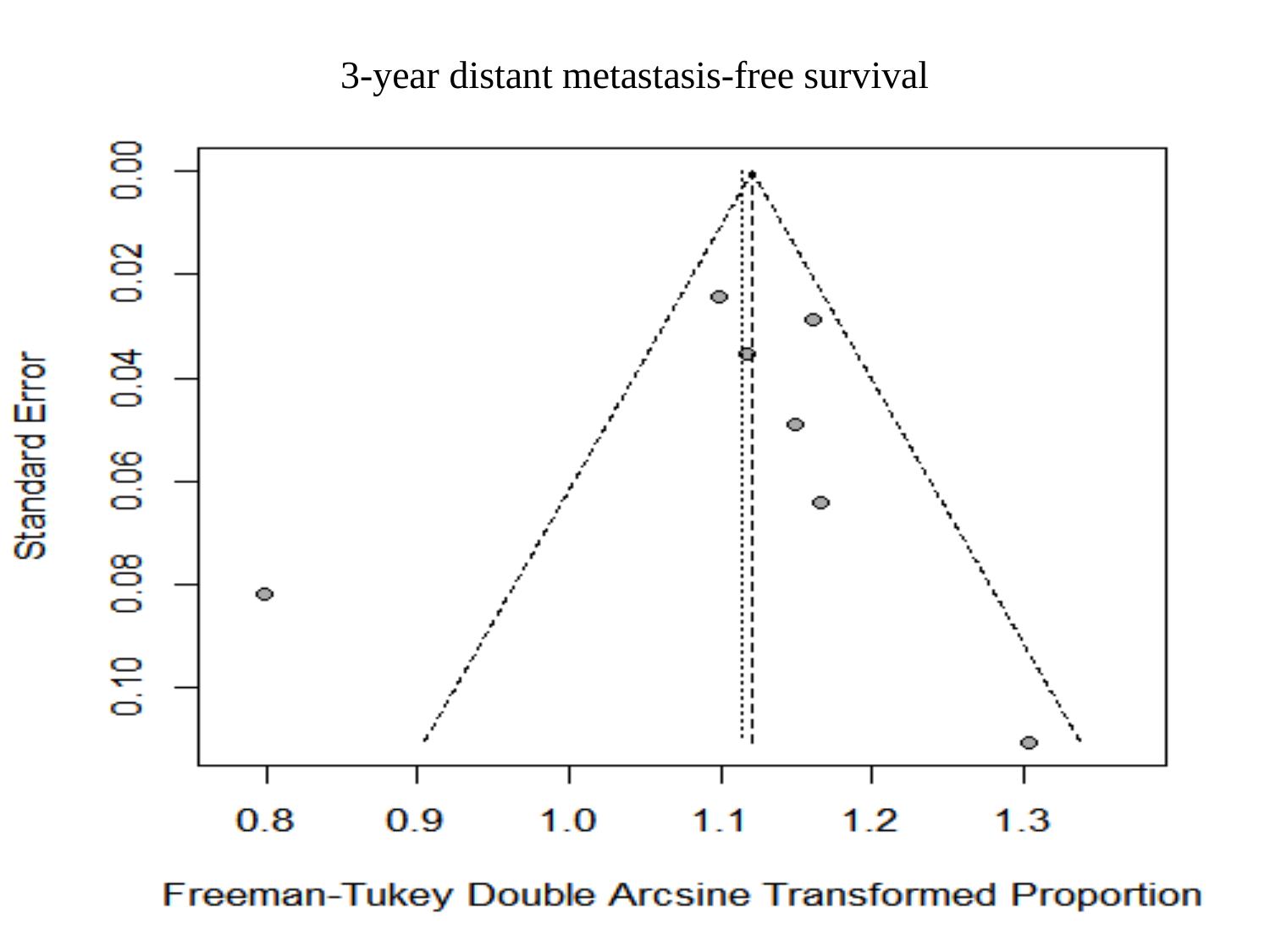

3-year distant metastasis-free survival

## Slide 5
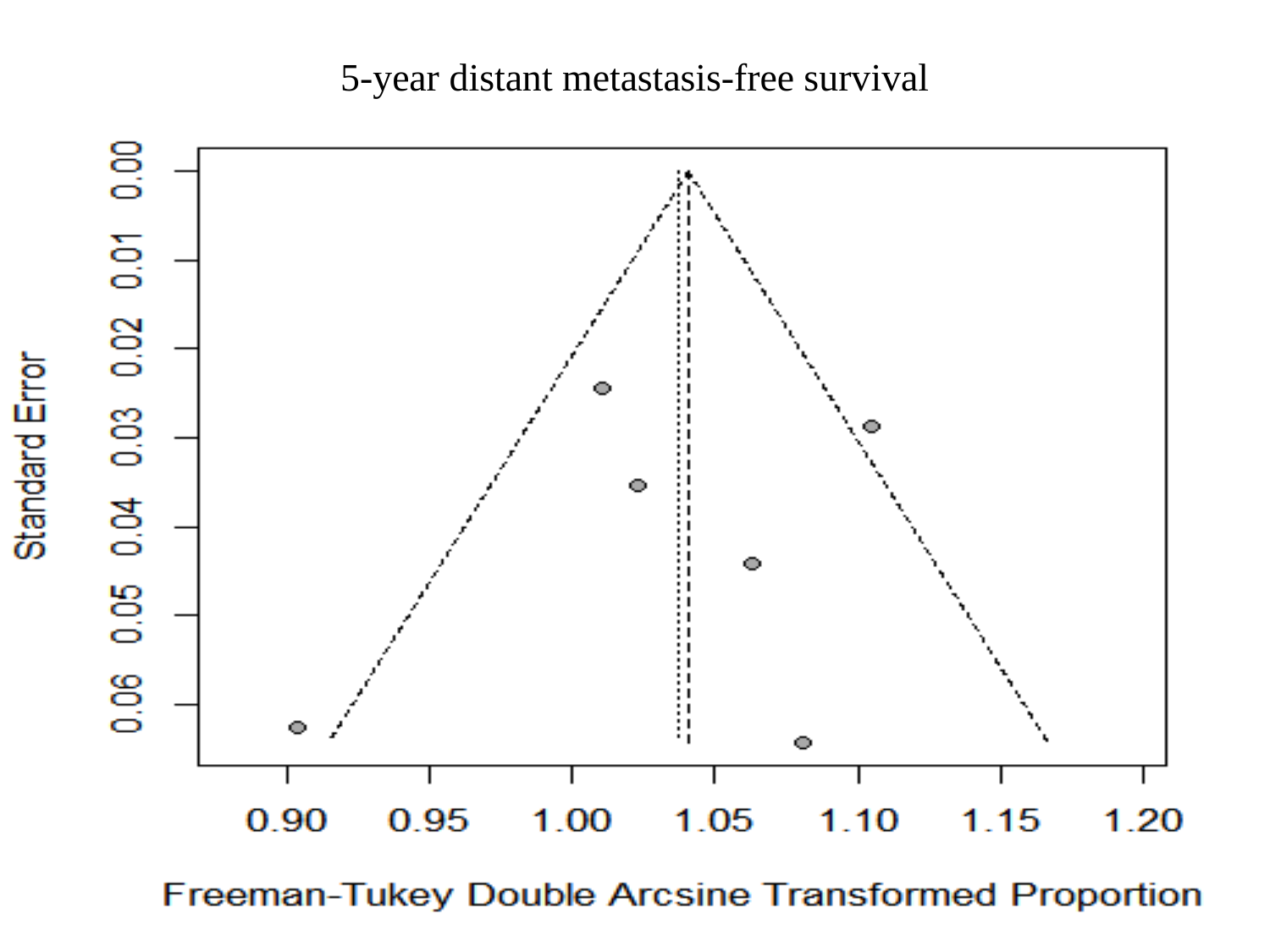

5-year distant metastasis-free survival

## Slide 6
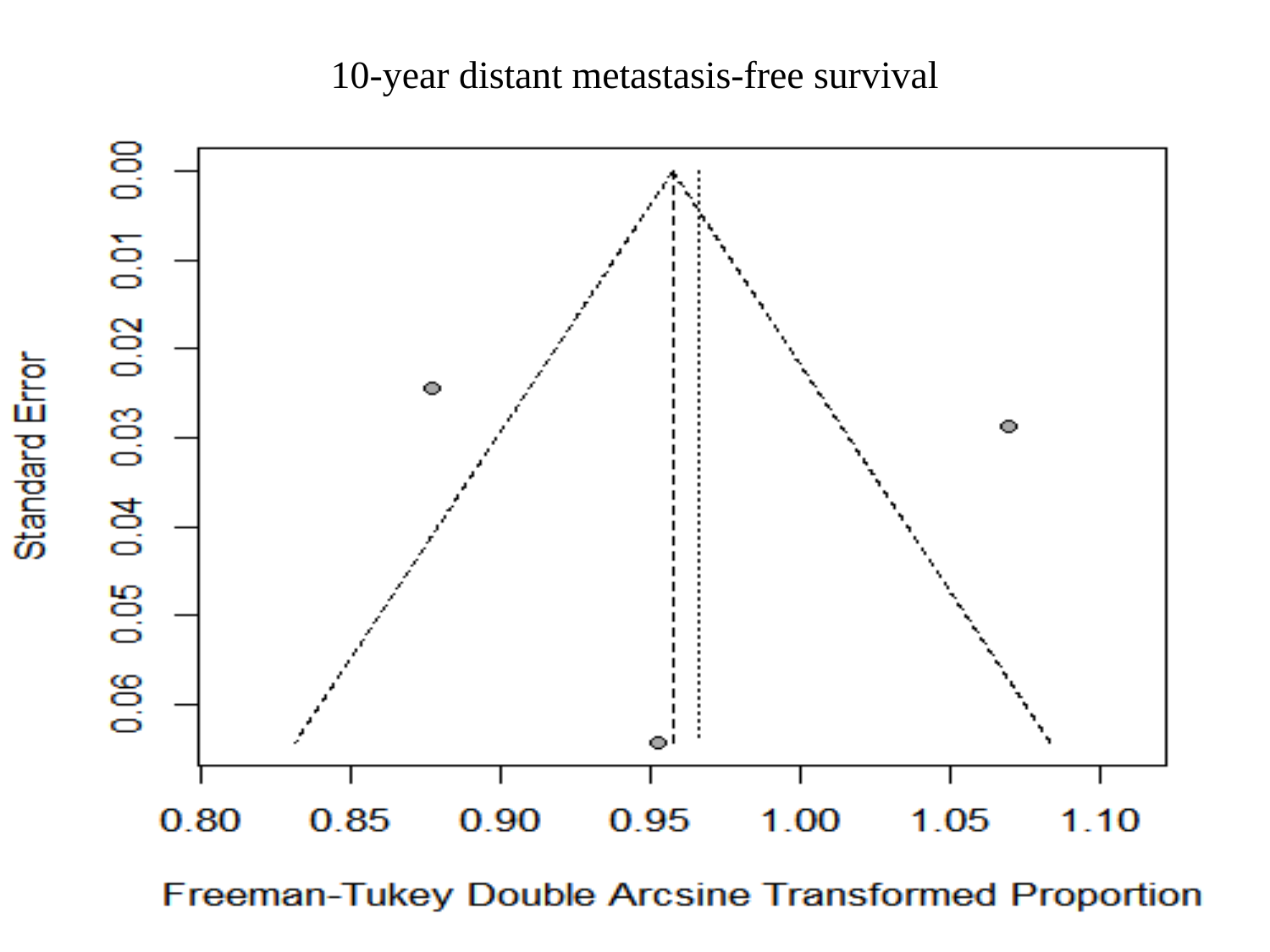

10-year distant metastasis-free survival

## Slide 7
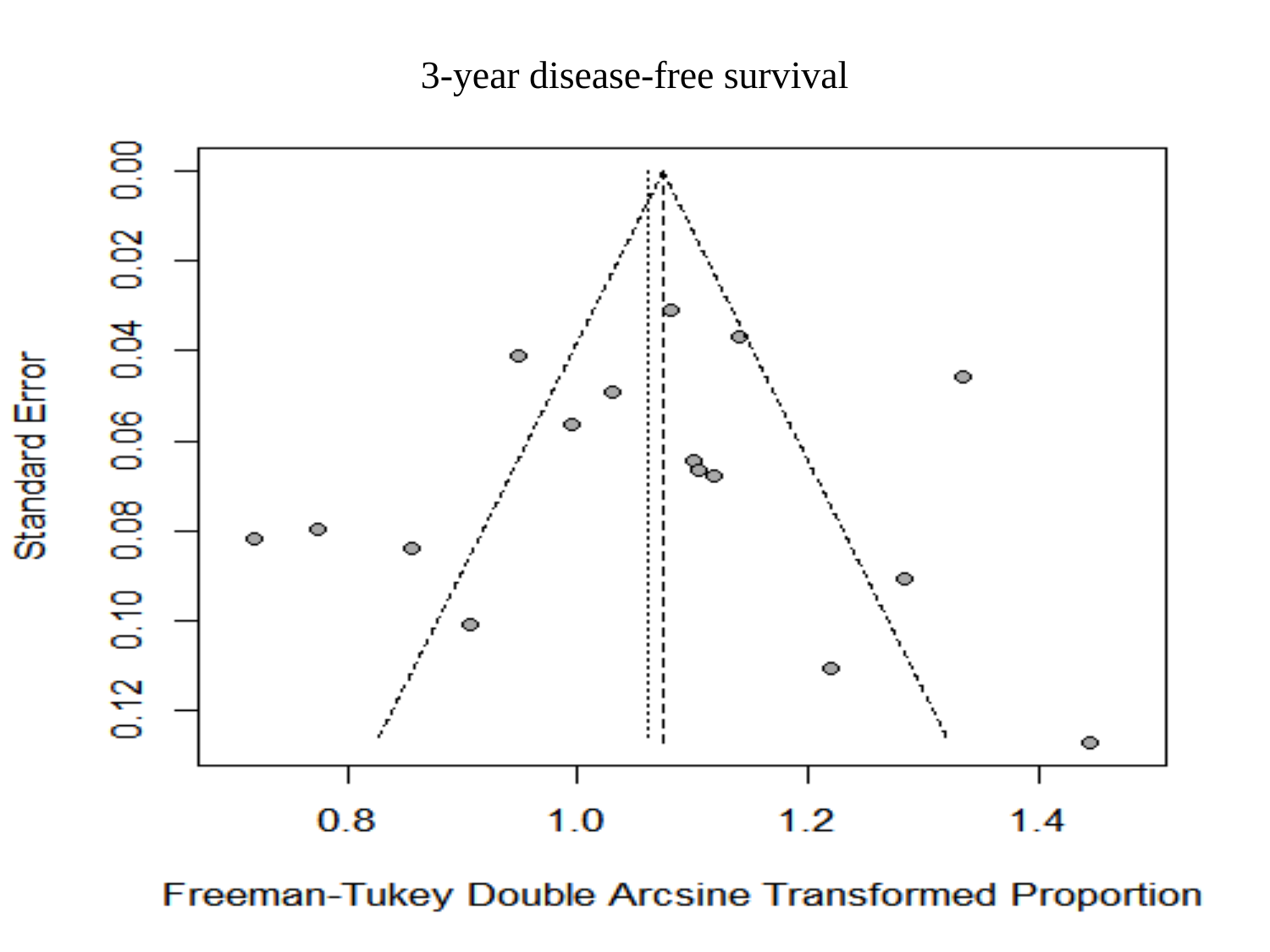

3-year disease-free survival

## Slide 8
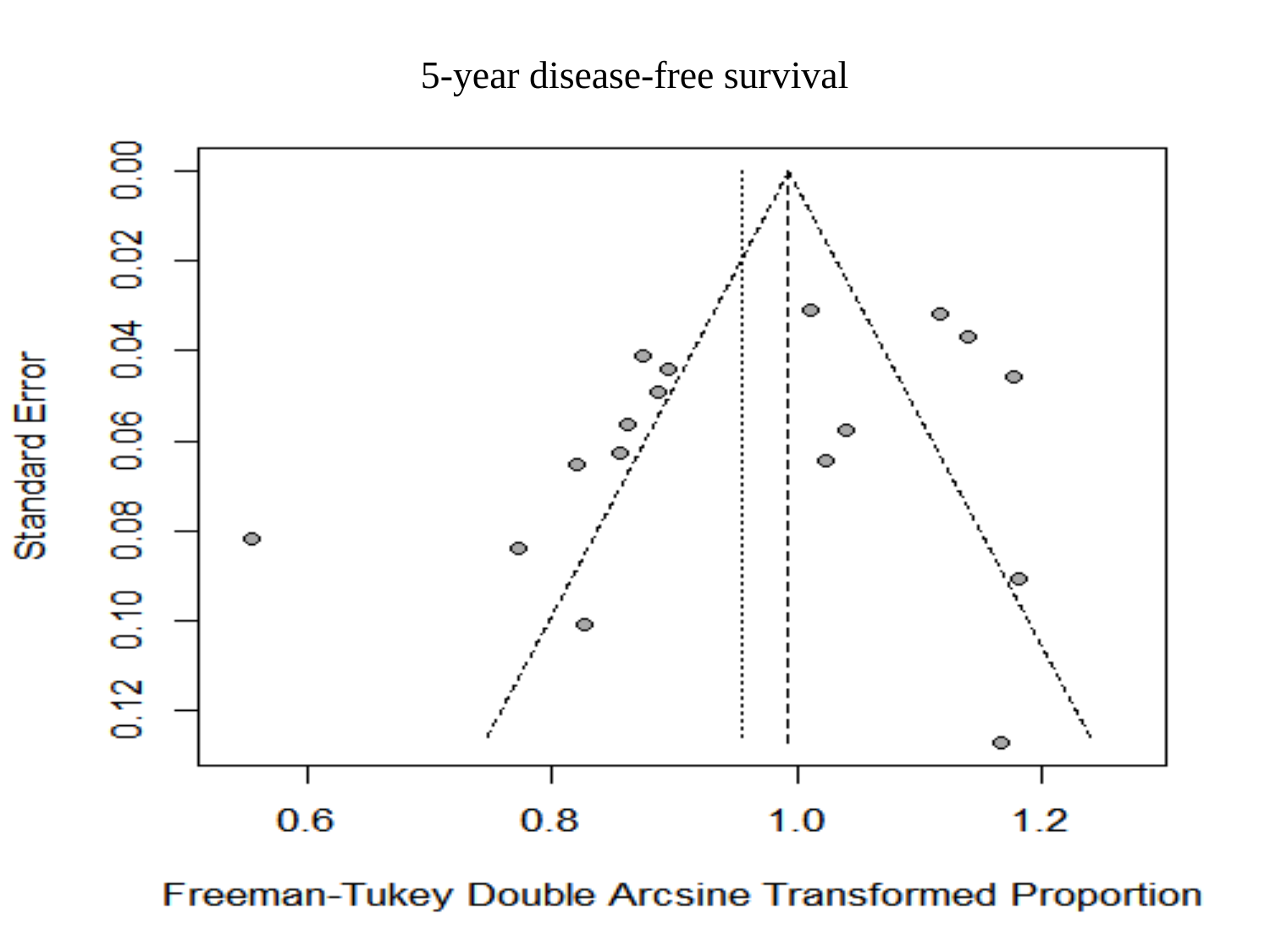

5-year disease-free survival

## Slide 9
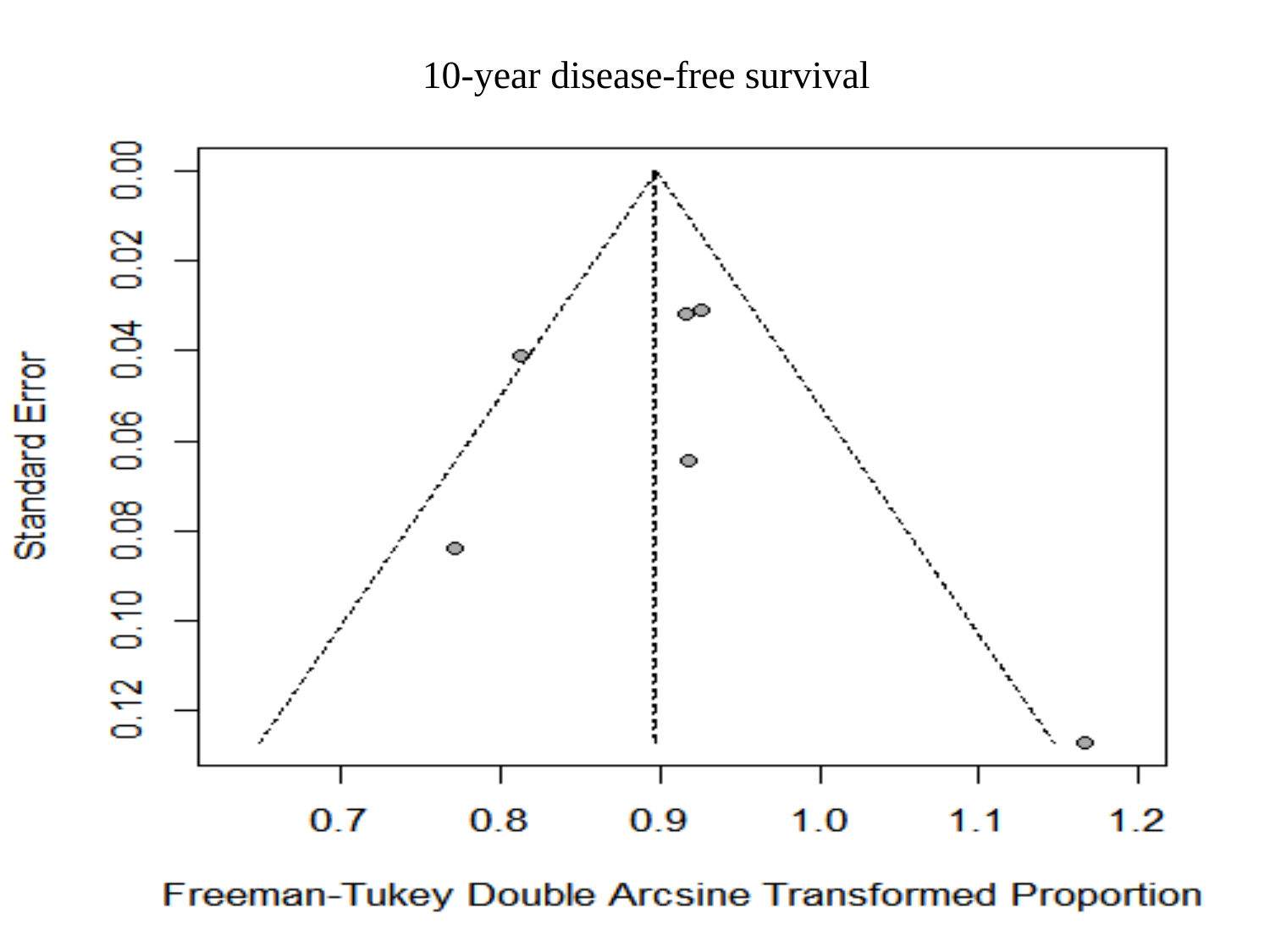

10-year disease-free survival

## Slide 10
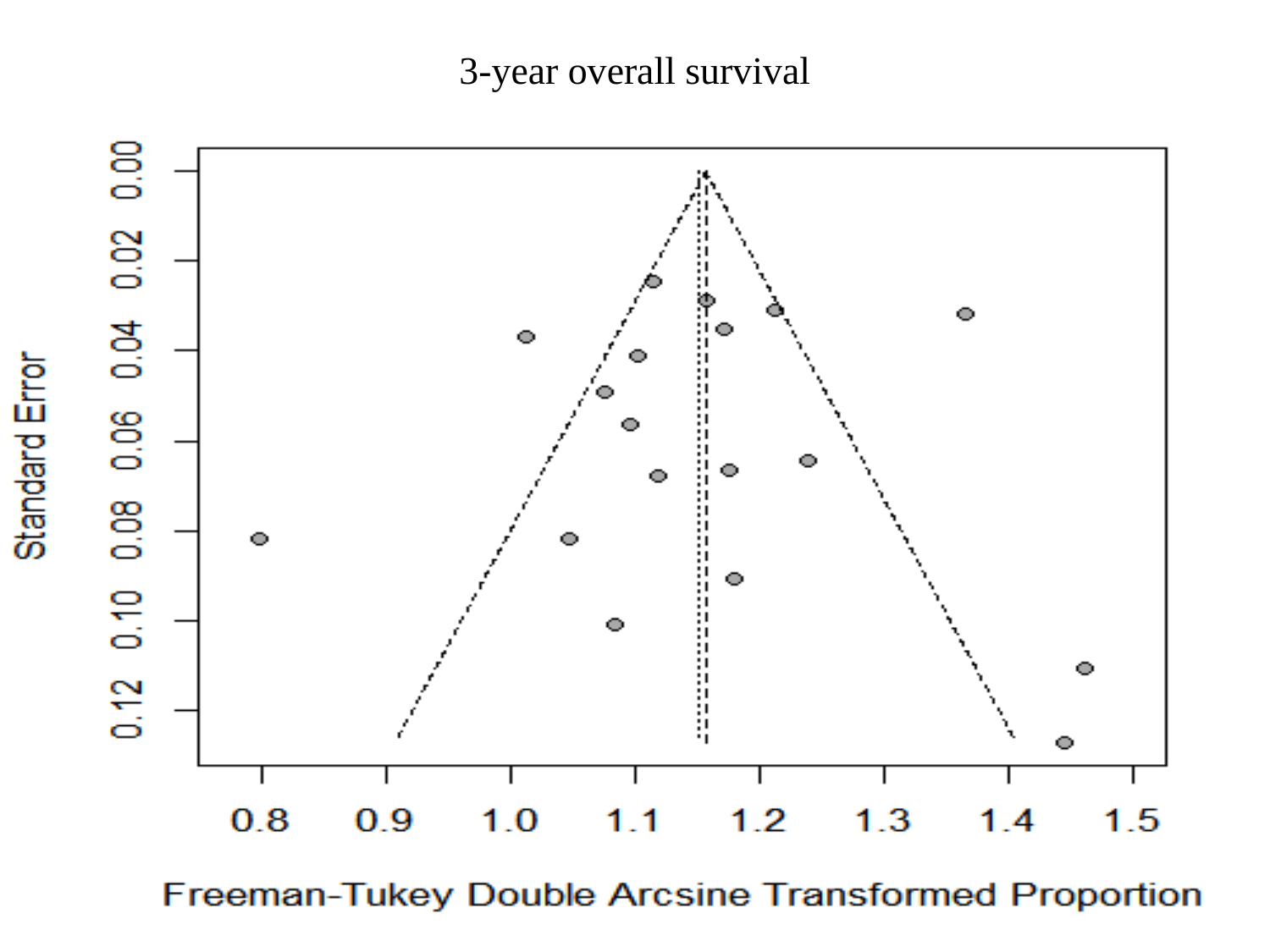

3-year overall survival

## Slide 11
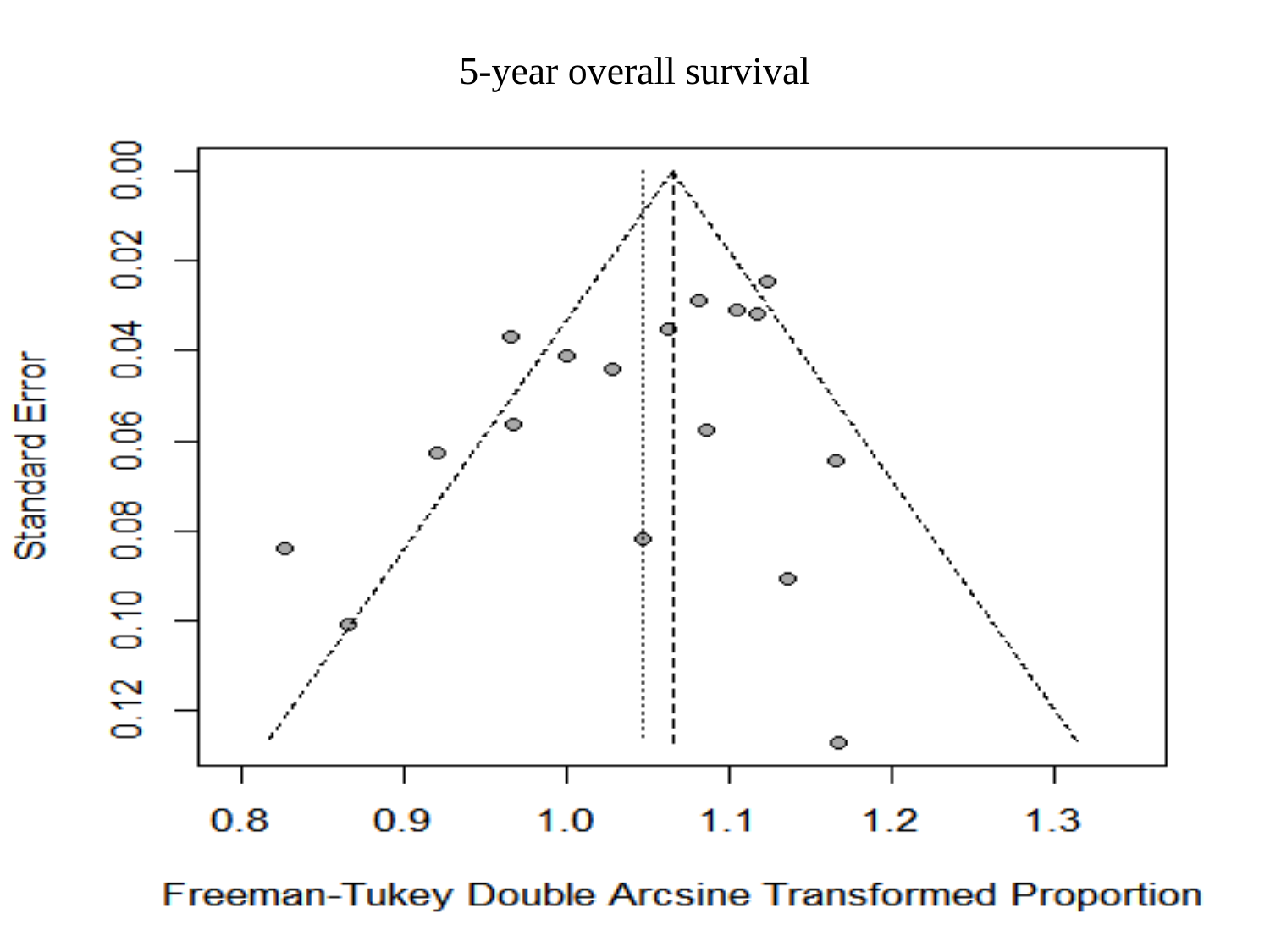

5-year overall survival

## Slide 12
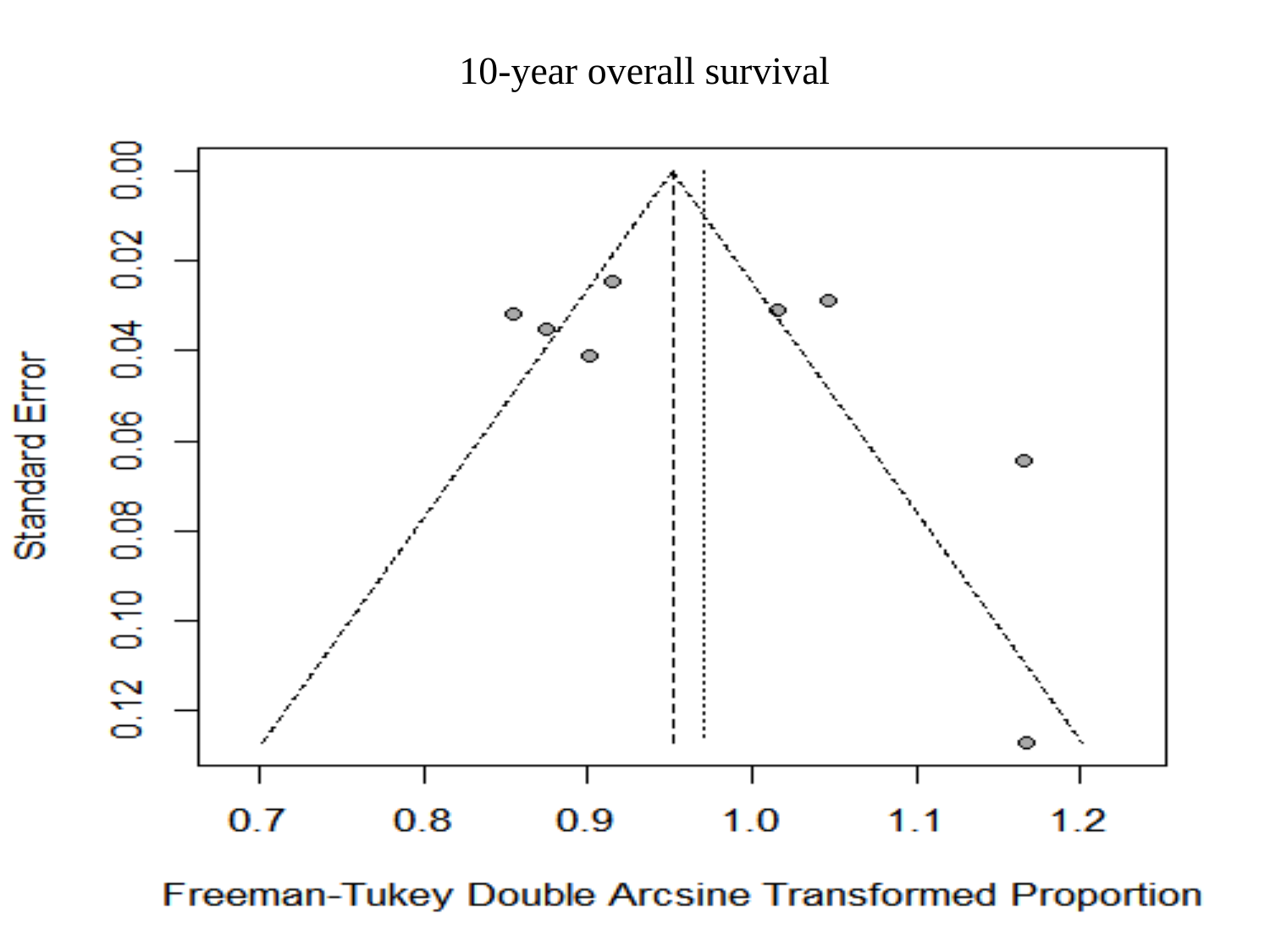

10-year overall survival
