# Supplementary material for: The Current Position of Postoperative Radiotherapy for Salivary Gland Cancer: A Systematic Review and Meta-Analysis
Source: Cancers (Basel). 2024 Jun 28;16(13):2375. doi: 10.3390/cancers16132375 (PMC11240508; doi:10.3390/cancers16132375)
Supplement: Supplementary file 1 [file cancers-16-02375-s001.zip › 20240519 supplementary Figure S2.pptx]

## Slide 1
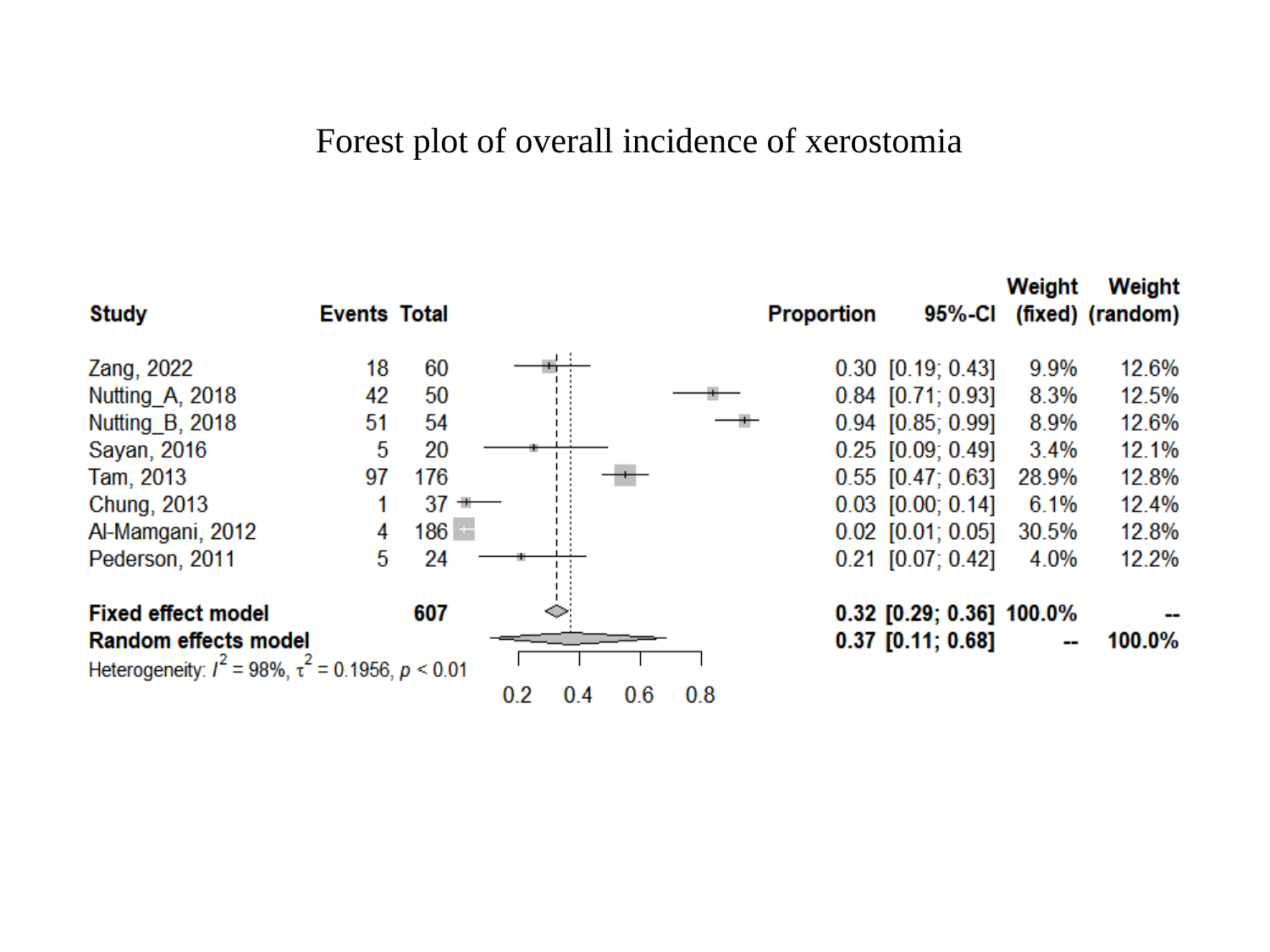

Forest plot of overall incidence of xerostomia

## Slide 2
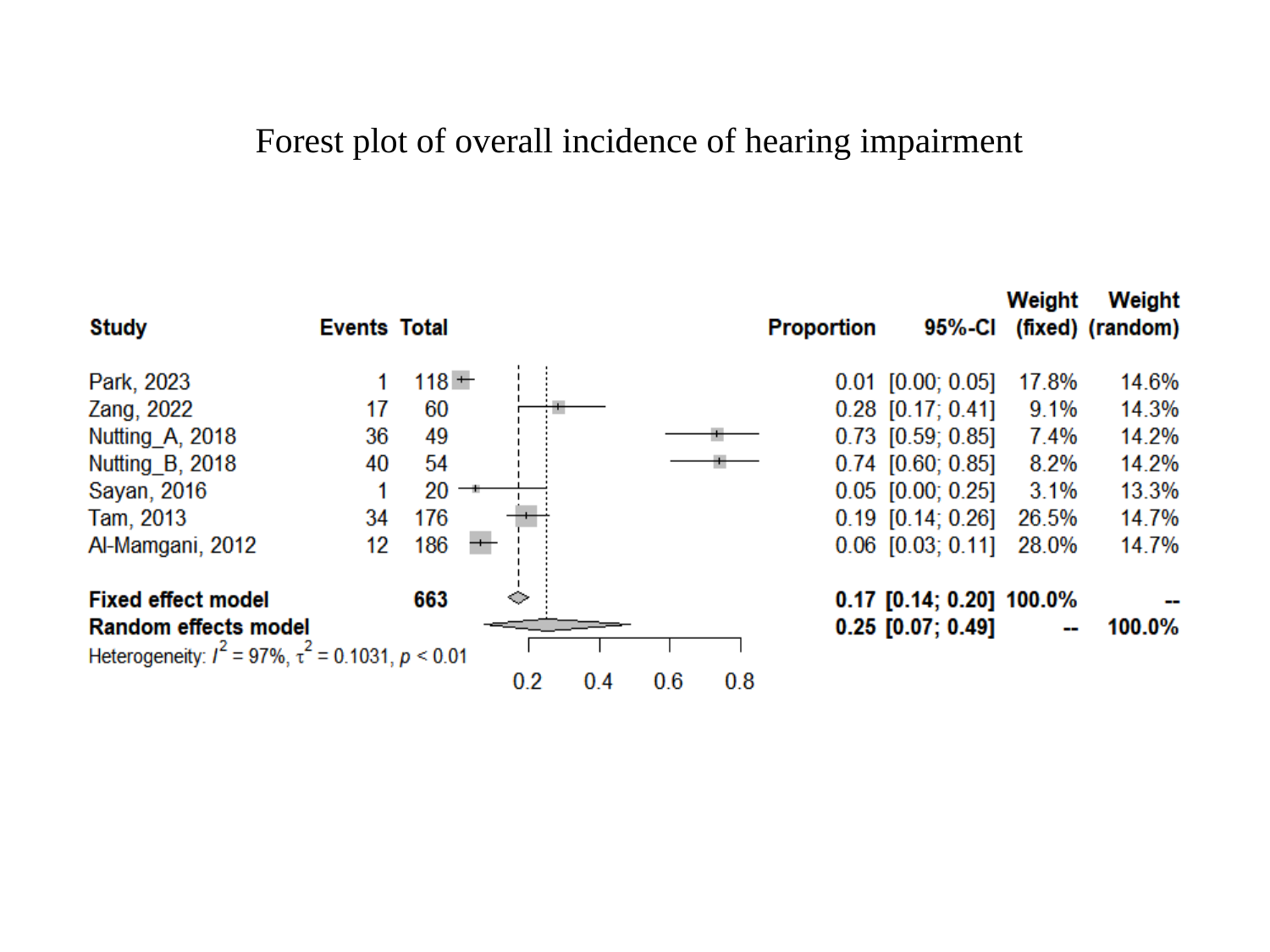

Forest plot of overall incidence of hearing impairment

## Slide 3
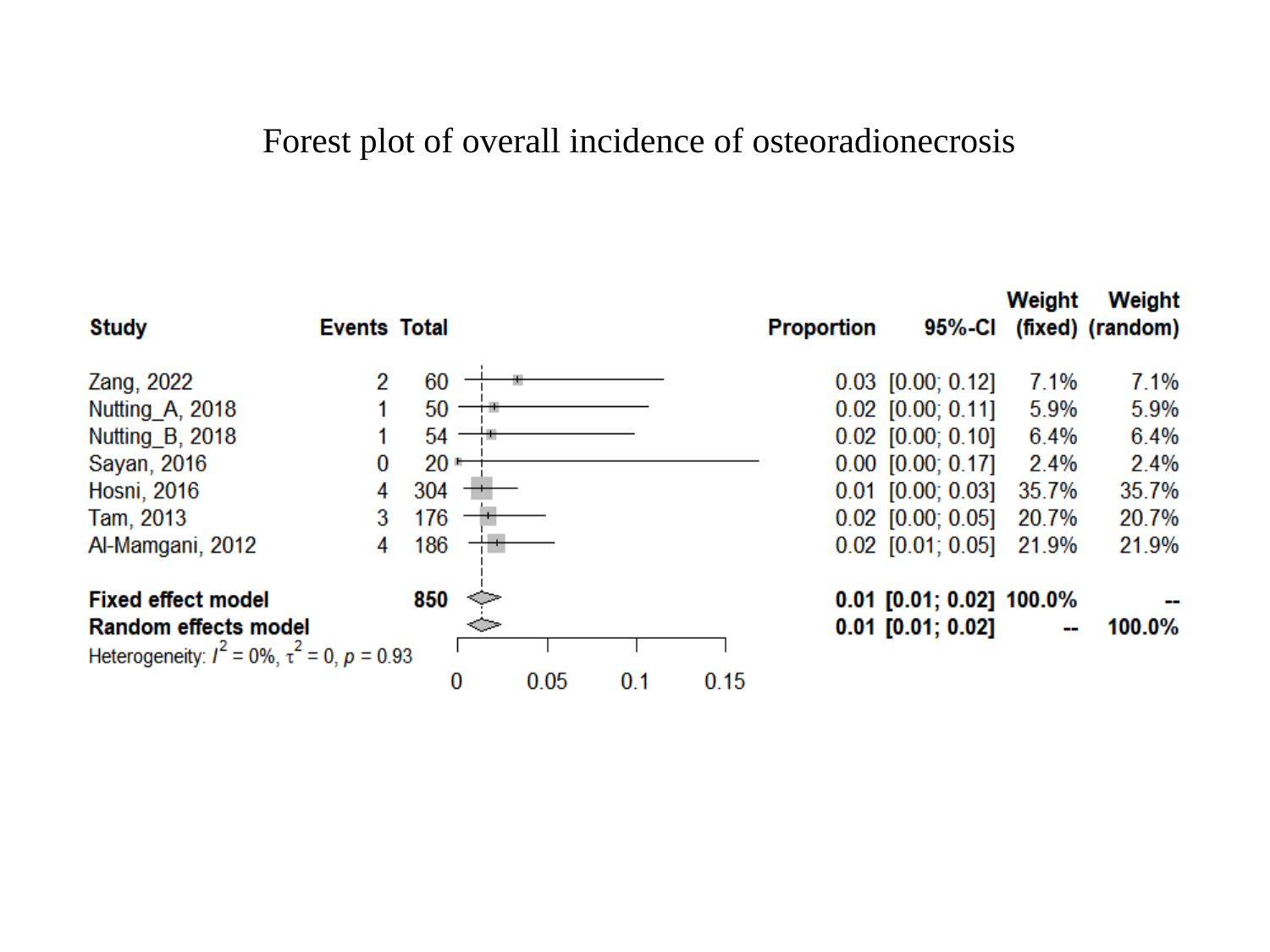

Forest plot of overall incidence of osteoradionecrosis

## Slide 4
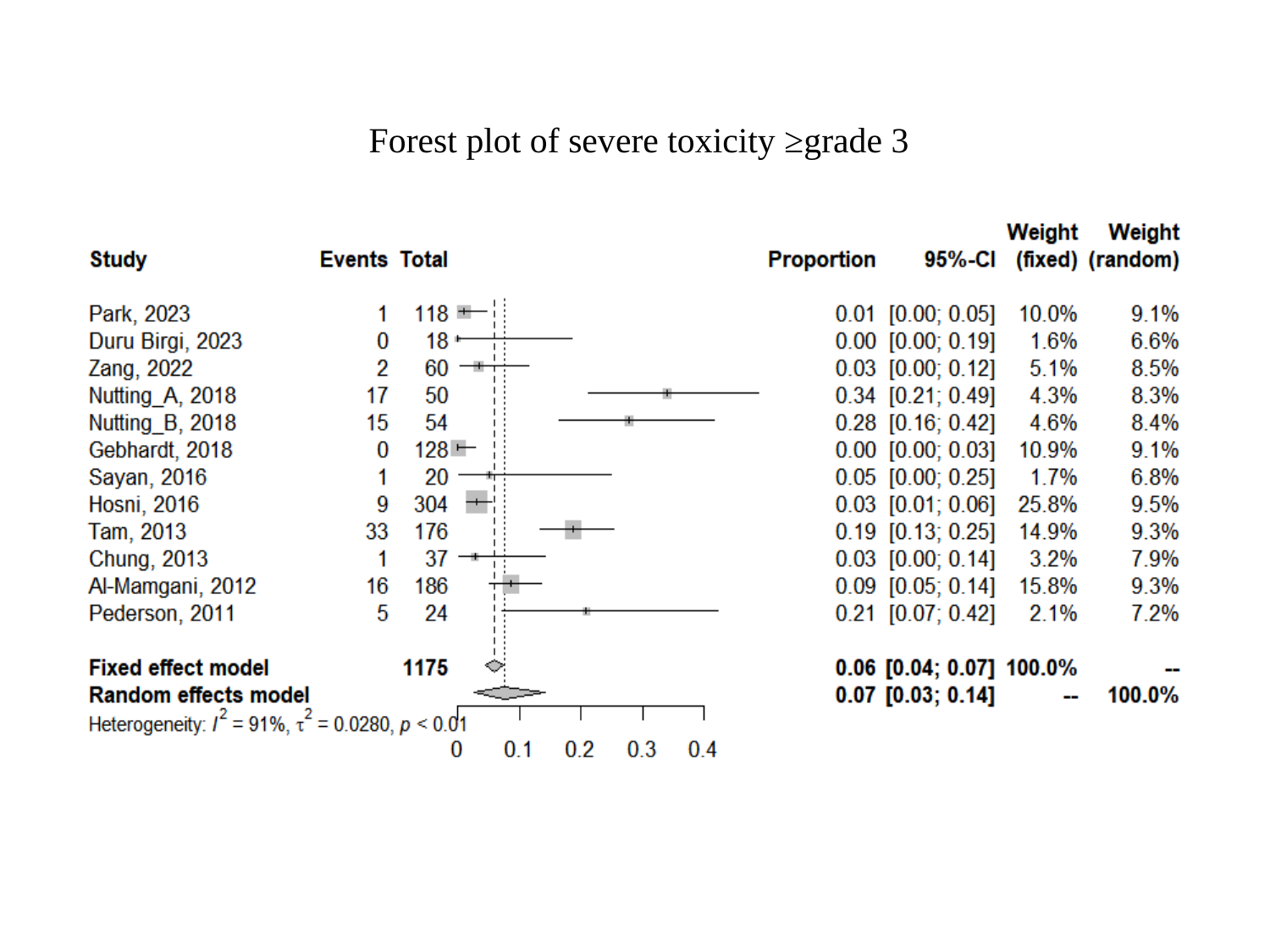

Forest plot of severe toxicity ≥grade 3
