# Supplementary material for: The Current Position of Postoperative Radiotherapy for Salivary Gland Cancer: A Systematic Review and Meta-Analysis
Source: Cancers (Basel). 2024 Jun 28;16(13):2375. doi: 10.3390/cancers16132375 (PMC11240508; doi:10.3390/cancers16132375)
Supplement: Supplementary file 1 [file cancers-16-02375-s001.zip › 20240620 supplementary table.docx]

Supplementary table 1**.** Search strategy and results

**(1) Keywords**

| **PICO** | **Fields** | **Keywords** | **Remarks** |
| --- | --- | --- | --- |
| **P** | MeSH | Salivary Glands | A |
|  |  | Salivary Glands, Minor |  |
|  |  | Parotid Gland |  |
|  |  | Submandibular Gland |  |
|  |  | Sublingual Gland |  |
|  | TIAB | Salivary Gland* |  |
|  |  | Parotid |  |
|  |  | Submandibular Gland* |  |
|  |  | Submaxillary Gland* |  |
|  |  | Sublingual Gland* |  |
|  |  | Saliva Gland* |  |
|  | MeSH | Neoplasms | B |
|  |  | Carcinoma |  |
|  |  | Adenocarcinoma |  |
|  | TIAB | Neoplasm* |  |
|  |  | Malignan* |  |
|  |  | Neoplasia* |  |
|  |  | Cancer* |  |
|  |  | Tumour* |  |
|  |  | Tumor* |  |
|  |  | Carcinoma* |  |
|  |  | Adenocarcinoma* |  |
|  |  | **A AND B** | C |
|  | MeSH | Salivary Gland Neoplasms | D |
|  |  | Submandibular Gland Neoplasms |  |
|  |  | Sublingual Gland Neoplasms |  |
|  |  | Parotid Neoplasms |  |
|  |  | **C OR D** | E |
| **I** | MeSH | Radiotherapy | F |
|  |  | Radiotherapy, Computer-Assisted |  |
|  |  | Radiotherapy, Intensity-Modulated |  |
|  |  | Radiotherapy, adjuvant |  |
|  |  | Radiotherapy, Image-Guided |  |
|  |  | Radiation |  |
|  | TIAB | Radiotherap* |  |
|  |  | Radiation* |  |
|  |  | Irradiation* |  |
|  |  | Volumetric Modulated Arc |  |
|  |  | Intensity Modulated Arc |  |
|  |  | Helical Tomotherap* |  |
|  |  | IMRT |  |
|  |  | EBRT |  |
|  |  | **E AND F** |  |

**(2) Search strategy**

| **DB** | **Search Strategy** |
| --- | --- |
| **PubMed** | ((("Salivary Glands"[MeSH Terms] OR "salivary glands, minor"[MeSH Terms] OR "Parotid Gland"[MeSH Terms] OR "Submandibular Gland"[MeSH Terms] OR "Sublingual Gland"[MeSH Terms] OR ("salivary gland*"[Title/Abstract] OR "Parotid"[Title/Abstract] OR "submandibular gland*"[Title/Abstract] OR "submaxillary gland*"[Title/Abstract] OR "sublingual gland*"[Title/Abstract] OR "saliva gland*"[Title/Abstract])) AND ("Neoplasms"[MeSH Terms] OR "Carcinoma"[MeSH Terms] OR "Adenocarcinoma"[MeSH Terms] OR ("neoplasm*"[Title/Abstract] OR "malignan*"[Title/Abstract] OR "neoplasia*"[Title/Abstract] OR "cancer*"[Title/Abstract] OR "tumour*"[Title/Abstract] OR "tumor*"[Title/Abstract] OR "carcinoma*"[Title/Abstract] OR "adenocarcinoma*"[Title/Abstract]))) OR ("Salivary Gland Neoplasms"[MeSH Terms] OR "Submandibular Gland Neoplasms"[MeSH Terms] OR "Sublingual Gland Neoplasms"[MeSH Terms] OR "Parotid Neoplasms"[MeSH Terms])) AND ("Radiotherapy"[MeSH Terms] OR "radiotherapy, computer assisted"[MeSH Terms] OR "radiotherapy, intensity modulated"[MeSH Terms] OR "radiotherapy, adjuvant"[MeSH Terms] OR "radiotherapy, image guided"[MeSH Terms] OR "Radiation"[MeSH Terms] OR ("radiotherap*"[Title/Abstract] OR "radiation*"[Title/Abstract] OR "irradiation*"[Title/Abstract] OR "Volumetric Modulated Arc"[Title/Abstract] OR "Intensity Modulated Arc"[Title/Abstract] OR "helical tomotherap*"[Title/Abstract] OR "IMRT"[Title/Abstract] OR "EBRT"[Title/Abstract])) |
| **EMBASE** | (((('salivary gland'/exp OR 'minor saliva gland'/exp OR 'parotid gland'/exp OR 'submandibular gland'/exp OR 'sublingual gland'/exp) OR ('salivary gland*':ab,ti OR 'parotid':ab,ti OR 'submandibular gland*':ab,ti OR 'submaxillary gland*':ab,ti OR 'sublingual gland*':ab,ti OR 'saliva gland*':ab,ti)) AND (('neoplasm'/exp OR 'carcinoma'/exp OR 'adenocarcinoma'/exp OR 'malignant neoplasm'/exp) OR ('Neoplasm*':ab,ti OR 'Malignan*':ab,ti OR 'Neoplasia*':ab,ti OR 'Cancer*':ab,ti OR 'Tumour*':ab,ti OR 'Tumor*':ab,ti OR 'Carcinoma*':ab,ti OR 'Adenocarcinoma*':ab,ti))) OR ('salivary gland tumor'/exp OR 'parotid gland tumor'/exp)) AND (('radiotherapy'/exp OR 'computer assisted radiotherapy'/exp OR 'intensity modulated radiation therapy'/exp OR 'adjuvant radiotherapy'/exp OR 'image guided radiotherapy'/exp OR 'radiation'/exp OR 'cancer radiotherapy'/exp OR 'irradiation'/exp OR 'volumetric modulated arc therapy'/exp OR 'intensity modulated radiation therapy'/exp OR 'tomotherapy'/exp OR 'external beam radiotherapy'/exp) OR ('radiotherap*':ab,ti OR 'radiation*':ab,ti OR 'irradiation*':ab,ti OR 'volumetric modulated arc':ab,ti OR 'intensity modulated arc':ab,ti OR 'helical tomotherap*':ab,ti OR 'IMRT':ab,ti OR 'EBRT':ab,ti)) |
| **Cochrane** | ((((MeSH descriptor: [Salivary Glands] explode all trees OR MeSH descriptor: [Salivary Glands, Minor] explode all trees OR MeSH descriptor: [Parotid Gland] explode all trees OR MeSH descriptor: [Submandibular Gland] explode all trees OR MeSH descriptor: [Sublingual Gland] explode all trees) OR ((salivary NEXT gland*):ti,ab,kw OR (Parotid):ti,ab,kw OR (submandibular NEXT gland*):ti,ab,kw OR (submaxillary NEXT gland*):ti,ab,kw OR (sublingual NEXT gland*):ti,ab,kw OR (saliva NEXT gland*):ti,ab,kw)) AND ((MeSH descriptor: [Neoplasms] explode all trees OR MeSH descriptor: [Carcinoma] explode all trees OR MeSH descriptor: [Adenocarcinoma] explode all trees) OR ((neoplasm*):ti,ab,kw OR (malignan*):ti,ab,kw OR (neoplasia*):ti,ab,kw OR (cancer*):ti,ab,kw OR (tumour*):ti,ab,kw OR (tumor*):ti,ab,kw OR (carcinoma*):ti,ab,kw OR (adenocarcinoma*):ti,ab,kw))) OR (MeSH descriptor: [Salivary Gland Neoplasms] explode all trees OR MeSH descriptor: [Submandibular Gland Neoplasms] explode all trees OR MeSH descriptor: [Sublingual Gland Neoplasms] explode all trees OR MeSH descriptor: [Parotid Neoplasms] explode all trees)) AND ((MeSH descriptor: [Radiotherapy] explode all trees OR MeSH descriptor: [Radiotherapy, Computer-Assisted] explode all trees OR MeSH descriptor: [Radiotherapy, Intensity-Modulated] explode all trees OR MeSH descriptor: [Radiotherapy, Adjuvant] explode all trees OR MeSH descriptor: [Radiotherapy, Image-Guided] explode all trees OR MeSH descriptor: [Radiation] explode all trees) OR ((radiotherap*):ti,ab,kw OR (radiation*):ti,ab,kw OR (irradiation*):ti,ab,kw OR ("Volumetric Modulated Arc"):ti,ab,kw OR ("Intensity Modulated Arc"):ti,ab,kw OR (helical NEXT tomotherap*):ti,ab,kw OR (IMRT):ti,ab,kw OR (EBRT):ti,ab,kw)) |
| **Web of Science** | ((TI=("Salivary Gland*") OR TI=("Parotid") OR TI=("Submandibular Gland*") OR TI=("Submaxillary Gland*") OR TI=("Sublingual Gland*") OR TI=("Saliva Gland*") OR AB=("Salivary Gland*") OR AB=("Parotid") OR AB=("Submandibular Gland*") OR AB=("Submaxillary Gland*") OR AB=("Sublingual Gland*") OR AB=("Saliva Gland*")) AND (TI=("Neoplasm*") OR TI=("Malignan*") OR TI=("Neoplasia*") OR TI=("Cancer*") OR TI=("Tumour*") OR TI=("Tumor*") OR TI=("Carcinoma*") OR TI=("Adenocarcinoma*") OR AB=("Neoplasm*") OR AB=("Malignan*") OR AB=("Neoplasia*") OR AB=("Cancer*") OR AB=("Tumour*") OR AB=("Tumor*") OR AB=("Carcinoma*") OR AB=("Adenocarcinoma*"))) AND (TI=("Radiotherap*") OR TI=("Radiation*") OR TI=("Irradiation*") OR TI=("Volumetric Modulated Arc") OR TI=("Intensity Modulated Arc") OR TI=("Helical Tomotherap*") OR TI=("IMRT") OR TI=("EBRT") OR AB=("Radiotherap*") OR AB=("Radiation*") OR AB=("Irradiation*") OR AB=("Volumetric Modulated Arc") OR AB=("Intensity Modulated Arc") OR AB=("Helical Tomotherap*") OR AB=("IMRT") OR AB=("EBRT")) |

**(3) Summary of search results**

| **No** | **DB** | **Results** | **Duplication** |
| --- | --- | --- | --- |
| 1 | PubMed (Medline) | 1,399 | 1,465 |
| 2 | EMBASE | 2,987 |  |
| 3 | Cochrane Library | 373 |  |
| 4 | Web of Science | 405 |  |
| Number of Search results (with duplication) | | 5,164 |  |
| **Number of Search results (without duplication)** | | **3,699** |  |

Supplementary table 2. Tumor details for salivary gland carcinoma treated with surgery followed by postoperative radiotherapy

| Author | Anatomical site (%) | Histology (%) | Positive/negative RM (%) | Definition of close RM | Definition of positive RM |
| --- | --- | --- | --- | --- | --- |
| Yan [17] | Major salivary glands (100)  - Parotid gland (75)  - Submandibular gland (24)  - Sublingual gland (1)  Minor salivary glands (0) | All subtypes (NS) | 4/96 | NS | NS |
| Park [18] | Major salivary glands (100)  - Parotid gland (100)  - Submandibular gland (0)  - Sublingual gland (0)  Minor salivary glands (0) | All subtypes (NS) | 36/64 | <1mm | Positive |
| Duru Birgi [19] | Major salivary glands (100)  - Parotid gland (100)  - Submandibular gland (0)  - Sublingual gland (0)  Minor salivary glands (0) | Mucoepidermoid carcinoma (28)/ Pleomorphic adenoma (22)/ Asinic cell carcinom (22)/ Adenocarcinoma (11)/ Myoepithelial carcinoma (11)/ Squamous cell carcinoma (6) | 22/78 | NS | NS |
| Hsieh_A [20] | Major salivary glands (77)  - Parotid gland (51)  - Submandibular gland (23)  - Sublingual gland (3)  Minor salivary glands (23) | Adenoid cystic carcinoma (40)/ Mucopeidermoid carcinoma (23)/ Carcinoma ex pleomorhpic adenoma (13)/ Acinic cell carcinoma (9)/ Adenocarcinoma (5)/ Lymphoepithelial carcinoma (4)/ Salivary duct carcinoma (3)/ Squamous cell carcinoma (2)/ Myoepithelial carcinoma (1)/ Adenosquamous carcinoma (0) | 70/30 | NS | <1mm s GRT |
| Hsieh_B [20] | Major salivary glands (72)  - Parotid gland (43)  - Submandibular gland (23)  - Sublingual gland (6)  Minor salivary glands (28) | Adenoid cystic carcinoma (29)/ Mucopeidermoid carcinoma (20)/ Lymphoepithelial carcinoma (15)/ Carcinoma ex pleomorhpic adenoma (8)/ Adenocarcinoma (9)/ Salivary duct carcinoma (6)/ Squamous cell carcinoma (5)/ Acinic cell carcinoma (4)/ Myoepithelial carcinoma (3)/ Poorly differentiated carcinoma (1) | 80/20 | NS | <1mm s GRT |
| Zang [21] | Major salivary glands (100)  - Parotid gland (57)  - Submandibular gland (28)  - Sublingual gland (15)  Minor salivary glands (0) | Adenoid cystic carcinoma (35)/ Lymphoepithelioid carcinoma (20)/ Mucoepidermoid carcinoma (13)/ Salivary duct carcinoma (8)/ Squamous cell carcinoma (7)/ Basal cell adenocarcinoma (5)/ Myoepithelial carcinoma (3)/ Acinic cell carcinoma (2)/ Others (7) | 12/88 | NS | NS |
| Franco [22] | Major salivary glands (94)  - Parotid gland (86)  - Submandibular gland (8)  - Sublingual gland (0)  Minor salivary glands (4) | All subtypes excluding adenoid cystic carcinoma (NS) | - | NS | NS |
| Dou [23] | Major salivary glands (60)  - Parotid gland (40)  - Submandibular gland (18)  - Sublingual gland (2)  Minor salivary glands (40) | Intermediate or high-grade histology (NS) | - | ≤5mm | Positive |
| Nutting_A [24] | Major salivary glands (100)  - Parotid gland (100)  - Submandibular gland (0)  - Sublingual gland (0)  Minor salivary glands (0) | All subtypes (NS) | 87/11 | NS | 1-5mm |
| Nutting_B [24] | Major salivary glands (100)  - Parotid gland (100)  - Submandibular gland (0)  - Sublingual gland (0)  Minor salivary glands (0) | All subtypes (NS) | 93/5 | NS | 1-5mm |
| Nishikado [25] | Major salivary glands (100)  - Parotid gland (100)  - Submandibular gland (0)  - Sublingual gland (0)  Minor salivary glands (0) | All subtypes (NS) | - | NS | NS |
| Li [26] | Major salivary glands (30)  - Parotid gland (25)  - Submandibular gland (5)  - Sublingual gland (0)  Minor salivary glands (70) | Intermediate-high grade histology:  Mucoepidermoid carcinoma (40)/ Adenoid cystic carcinoma (20)/ Carcinoma ex pleomorphic adenoma (20)/ Poorly differentiated adenocarcinoma (10)/ Others (10) | 5/95 | ≤1mm | Positive |
| Gebhardt [27] | Major salivary glands (94)  - Parotid gland (81)  - Submandibular gland (13)  - Sublingual gland (0)  Minor salivary glands (6) | Mucoepidermoid carcinoma (19)/ Salivary duct carinoma (19)/ Adenoid cystic carcinoma (18)/ Adenocarcinoma (13)/ Acinic cell carcinoma (11)/ Epithelial myoepithelial carcinoma (10)/ Carcinoma ex pleomorphic adenoma (9)/ Lymphoepithelial carcinoma (1)/ | 53/43 | ≤1mm | Positive |
| Boon [28] | Major salivary glands (80)  - Parotid gland (73)  - Submandibular gland (7)  - Sublingual gland (0)  Minor salivary glands (20) | Secretory carcinoma with ETV6-NTRK3 fusion gene (100) | 47/53 | 1-5mm | <1mm |
| Zhang [29] | Major salivary glands (100)  - Parotid gland (100)  - Submandibular gland (0)  - Sublingual gland (0)  Minor salivary glands (0) | Mucoepideromoid carcinoma (41)/ Adenoid cystic carcinoma (24)/ Acinic cell carcinoma (14)/ Nonspecific poorly differentiated adenocarcinoma (3)/ Salivary duct carcinoma (3)/ Malignancy of pleomorphic adenoma (3)/ Lymphoepithelial carcinoma (3)/ Epithelial myoepithelial carcinoma (3)/ Carcinoma ex pleomorphic adenoma (3)/ Basal cell carcinoma (3) | - | NS | NS |
| Gutschenritter [30] | Major salivary glands (NR)  - Parotid gland (NR)  - Submandibular gland (NR)  - Sublingual gland (NR)  Minor salivary glands (NR) | All subtypes (NS) | - | NS | NS |
| Sayan [31] | Major salivary glands (100)  - Parotid gland (85)  - Submandibular gland (15)  - Sublingual gland (0)  Minor salivary glands (0) | Mucoepidermoid carcinoma (45)/ Adenoid cystic carcinoma (25)/ Others (30) | 45/55 | <5mm | Positive |
| Mifsud_A [32] | Major salivary glands (60)  - Parotid gland (NR)  - Submandibular gland (NR)  - Sublingual gland (NR)  Minor salivary glands (40) | Adenoid cystic carcinoma (29)/ Mucoepidermoid carcinoma (26)/ Adenocarcinoma (22)/ Acinic cell carcinoma (12)/ Salivary duct carcinoma (8)/ others (8) | 41/59 | NS | NS |
| Mifsud_B [32] | Major salivary glands (78)  - Parotid gland (NR)  - Submandibular gland (NR)  - Sublingual gland (NR)  Minor salivary glands (22) | Adenoid cystic carcinoma (35)/ Mucoepidermoid carcinoma (24)/ Salivary duct carcinoma (24)/ Adenocarcinoma (17) | 78/22 | NS | NS |
| Hosni [33] | Major salivary glands (100)  - Parotid gland (78)  - Submandibular gland (21)  - Sublingual gland (1)  Minor salivary glands (0) | Mucoepidermoid carcinoma (18)/ Adenoid cystic carcinoma (18)/ Acinic cell carcinoma (16)/ Salivary duct carcinoma (13)/ Carcinoma ex pleomorphic adenoma (7)/ Adenocarcinoma (5)/ Squamous cell carcinoma (4)/ Others (19) | 50/45 | <5mm | Positive |
| Haderlein [34] | Major salivary glands (82)  - Parotid gland (65)  - Submandibular gland (17)  - Sublingual gland (0)  Minor salivary glands (18) | Adenoid cystic carcioma (35)/ Mucoepidermoid carcinoma (27)/ Ductal adenocarcinoma (18)/ Adenocarcinoma NOS (11)/ Acinic cell carcinoma (6)/ Others (3) | 11/81 | NS | NS |
| Kaur [35] | - Major salivary glands (NR)  - Parotid gland (NR)  - Submandibular gland (NR)  - Sublingual gland (NR)  Minor salivary glands (NR) | All subtypes (NS) | - | <5mm | Positive |
| Tam [36] | Major salivary glands (100)  - Parotid gland (84)  - Submandibular gland (16)  - Sublingual gland (1)  Minor salivary glands (0) | Adenocarcinoma (23)/ Mucoepidermoid carcinoma (25)/ Adenoid cystic carcinoma (16)/ Acinic cell carcinoma (15)/ Myoepithelial carcinoma (11)/ Salivary duct carcinoma (9) Poorly differentiated carcinoma (2) | 47/48 | NS | Positive |
| Chung [37] | Major salivary glands (100)  - Parotid gland (92)  - Submandibular gland (8)  - Sublingual gland (0)  Minor salivary glands (0) | Nonadenoid cystic carcinoma:  Adenocarcinoma (35)/ Squamous cell carcinoma (22)/ Mucoepidermoid carcinoma (22)/ Malignant mixed tumor (13)/ Poorly differentiated carcinoma (8) | 77/20 | NS | ≤3mm |
| Kim [38] | Major salivary glands (100)  - Parotid gland (63)  - Submandibular gland (34)  - Sublingual gland (3)  Minor salivary glands (0) | Salivary duct carcinoma (100) | 49/51 | NS | NS |
| Al-Mamgani [39] | Major salivary glands (100)  - Parotid gland (100)  - Submandibular gland (0)  - Sublingual gland (0)  Minor salivary glands (0) | Adenoid cystic carcinoma (25)/ Mucoepidermoid carcinoma (19)/ Acinic cell carcinoma (16)/ Squamous cell carcinoma (13)/ Adenocarcinoma (9)/ Carcinoma ex pleomorphic adenoma (5)/ Epithelial-myoepithelial carcinoma (5)/ Salivary duct carcinoma (3)/ Undifferentiated carcinoma (3)/ Others (2) | - | ≤5mm | NS |
| Pederson [40] | Major salivary glands (84)  - Parotid gland (71)  - Submandibular gland (13)  - Sublingual gland (0)  Minor salivary glands (16) | Adenoid cystic carcinoma (21)/ Mucoepidermoid carcinoma (21)/ Adenocarcinoma (13)/ Squamous cell carcinoma (13)/ Poorly differentiated carcinoma (13)/ Carcinoma ex pleomorphic adenoma (8)/ Myoepithelial carcinoma (4)/ Salivary duct carcinoma (4)/ Anaplastic carcinoma (4) | - | NS | NS |
| Noh [41] | Major salivary glands (100)  - Parotid gland (75)  - Submandibular gland (20)  - Sublingual gland (5)  Minor salivary glands (0) | Mucoepidermoid carcinoma (24)/ Adenoid cystic carcinoma (24)/ Salivary duct carcinoma (16)/ Carcinoma ex pleomorphic adenoma (13)/ Adenocarcinoma (11)/ Acinic cell carcinoma (3)/ Others (9) | 28/68 | <5mm | Positive |
| Chen [42] | Major salivary glands (55)  - Parotid gland (35)  - Submandibular gland (20)  - Sublingual gland (0)  Minor salivary glands (45) | Adenoid cystic carcinoma (33)/ Mucoepidermoid carcinoma (24)/ Adenocarcinoma (23)/ Acinic cell carcinoma (8)/ Undifferentiated carcinoma (4)/ Carcinoma ex pleomorphic adenoma (3)/ Squamous cell carcinoma (3)/ Salivary duct carcinoma (2) | 56/44 | NS | Positive |

NR, not reported; NS, not specified; RM, resection margin.

Supplementary table 3. Pooled rates of distant metastases free survival (DMFS)

| Group | Cohorts (n) | Patients (n) | p, Heterogeneity | I^2^ | Egger’s test, p | Fixed Event rate (95% CI) | Random Event rate (95% CI) | p (between groups) |
| --- | --- | --- | --- | --- | --- | --- | --- | --- |
| 3-year DMFS |  |  |  |  |  |  |  |  |
| ALL | 7 | 1142 | 0.0013 | 72.52% | 0.8200 | 0.82 (0.79-0.84) | 0.81 (0.76-0.86) |  |
| Postop CCRT^a^ | 2 | 57 | 0.0002 | 92.61% | - | 0.69 (0.57-0.81) | 0.76 (0.27-1.00) | 0.6908 |
| PORT^b^ | 1 | 103 | - | - | - | 0.83 (0.76-0.90) | 0.83 (0.76-0.90) |  |
| High grade >50%^c^ | 1 | 37 | - | - | - | 0.51 (0.35-0.67) | 0.51 (0.35-0.67) | < 0.0001 |
| High grade ≤50% | 4 | 845 | 0.1487 | 43.79% | 0.2312 | 0.82 (0.80-0.85) | 0.83 (0.79-0.87) |  |
| mRT dose >64 Gy | 2 | 324 | 0.2091 | 36.62% | - | 0.86 (0.81-0.89) | 0.87 (0.78-0.94) | 0.1603 |
| mRT dose ≤64 Gy | 4 | 400 | 0.0012 | 81.08% | 0.4082 | 0.80 (0.76-0.84) | 0.77 (0.66-0.87) |  |
| 5-year DMFS |  |  |  |  |  |  |  |  |
| ALL | 6 | 1173 | 0.0310 | 59.31% | 0.7235 | 0.75 (0.72-0.77) | 0.74 (0.70-0.79) |  |
| Postop CCRT | 0 | 0 | - | - | - | - | - | - |
| PORT | 0 | 0 | - | - | - | - | - |  |
| High grade >50% | 1 | 63 | - | - | - | 0.62 (0.50-0.74) | 0.62 (0.50-0.74) | 0.0294 |
| High grade ≤50% | 3 | 850 | 0.0399 | 68.95% | 0.7470 | 0.76 (0.73-0.78) | 0.76 (0.70-0.81) |  |
| mRT dose >64 Gy | 2 | 432 | 0.4237 | 0% | - | 0.79 (0.75-0.83) | 0.79 (0.75-0.83) | 0.0849 |
| mRT dose ≤64 Gy | 3 | 323 | 0.1206 | 52.72% | 0.8415 | 0.72 (0.67-0.77) | 0.72 (0.63-0.79) |  |
| 10-year DMFS |  |  |  |  |  |  |  |  |
| All | 3 | 782 | <0.0001 | 92.36% | 0.8862 | 0.67 (0.64-0.70) | 0.68 (0.54-0.80) |  |
| Postop CCRT | 0 | 0 | - | - | - | - | - | - |
| PORT | 0 | 0 | - | - | - | - | - |  |
| High grade >50% | 0 | 0 | - | - | - | - | - | - |
| High grade ≤50% | 2 | 722 | <0.0001 | 96.18% | - | 0.67 (0.63-0.70) | 0.68 (0.50-0.84) |  |
| mRT dose >64 Gy | 1 | 304 | - | - | - | 0.77 (0.72-0.82) | 0.77 (0.72-0.82) | 0.0970 |
| mRT dose ≤64 Gy | 1 | 60 | - | - | - | 0.67 (0.54-0.78) | 0.67 (0.54-0.78) |  |

CI, confidence interval; postop CCRT, postoperative concurrent chemoradiotherapy; PORT, postoperative radiotherapy; mRT dose, median radiotherapy dose.

^a^includes cohorts with all patients receiving postop CCRT.

^b^includes cohorts with all patients receiving PORT alone. Cohorts with mixed patients receiving postop CCRT or PORT were excluded.

^c^means that the proportion of the patients with high grade is beyond 50% among the entire patients.
